# On the Degeneracy of the Human Race
*"Traité des Dégenérescences physiques, intellectuelles, et morales de l'espéce humaine; et des causes qui produisent ces variétés maladives." Par le Docteur B. A. Morel. Paris. 1857.


**Published:** 1857-04-01

**Authors:** 


THE JOURNAL

OF

PSYCHOLOGICAL MEDICINE

AND

MENTAL PATHOLOGY.

APRIL 1, 1857.

### Part First.

### Original Communications.

### Art. I.—ON THE DEGENERACY OF THE HUMAN RACE.*

MAN alone is a cosmopolite; he alone inhabits the entire earth. Where the bear and the reindeer can scarce exist; where the lizard perishes parched with thirst; where the condor soars thousands of feet above the level of the sea; and hundreds of feet below the surface, where the rat hardly ekes out a precarious subsistence, there man finds a home and flourishes. But whilst he thus asserts his authority over nature, she in turn sets her seal upon him; and, according to the climate, the geological structure of the soil, and the ever-varying physical conditions with which he is surrounded, the primitive type becomes modified to produce the striking varieties in colour, form, and general physical, psychical, and moral development, which have been so often mistaken for irrefragable evidence of distinct origin. These are what are termed the *natural* modifications of the human race.

But under exceptional conditions the contest of man with the various elemental influences is partially unsuccessful, and he becomes *unnaturally* or *morbidly* removed from the primitive type. The same result is brought about by various circumstances attendant upon his nutrition, his social condition, his habits of life, hereditary influence, and many other causes. This constitutes a "*degeneration*"—defined by M. Morel as a "morbid

* "Traité des Dégénérescences physiques, intellectuelles, et morales de l'espèce humaine; et des causes qui produisent ces variétés maladives." Par le Docteur B. A. Morel. Paris. 1857.

deviation from a primitive type;" and characterized by a tendency to further deterioration, to hereditary transmission, and to the more or less speedy extinction of that section of the race or community. And as in the natural modifications of type, there are certain forms occurring with *constant* relation to the causes in operation in their production; so in the morbid deviations there are also certain forms, not occurring casually, irregularly, or interchangeably; but marked by definite characters, and bearing constant and definite relations to their causes.

It is not alone the fact that the nervous system, in its double connexion with mind and body, is most frequently the victim of these degenerations, that lends a deep interest to this inquiry in reference to psychology; but also that according to M. Morel, mental alienation in its various forms, but especially the chronic, is but the concentrated and final expression of degeneracy of race, wheresoever the chain of morbid phenomena commenced. "What," he asks, "are in reality the asylums for the insane, but the concentration of the principal degenerations of the human race?"*

Such is a brief abstract of the principles which receive illustration in this very able contribution to anthropology; their special and important bearing upon psychology and practical psychiatry will be seen from the following remarks contained in the preface:—

"My constant desire has been (and is) to complete my labours upon mental alienation by a system of therapeutics designed to make generally available the means to prevent and combat this cruel affection. I very soon discovered, however, that the question was much more vast and complex than I could have supposed. I was compelled, therefore, to enter more profoundly into the study of nervous affections, as much in relation to their causes, as to their pathological transformations.

"My actual conviction is, that the insane of our asylums are, in the majority of cases, only the representatives of certain morbid varieties of the race; modifiable, in some instances, irretrievable in others. Whatever may be the origin of their affections, they are all more or less marked with the characters of disease of long duration, in which dominates the redoubtable influence of hereditary transmission . . . .

"The incessant increase in Europe, not only of mental alienation, but of all those abnormal conditions which bear special relation to the existence of moral and physical evil in humanity, was also a striking fact in my investigations. Everywhere I heard physicians complaining of the increasing number of the insane, and of the more frequent complications which general paralysis, epilepsy, and a more marked depression of the intellectual and physical powers contributed to lessen the chances of cure.

"Add also, that neuroses, such as hysteria and hypochondria, frequently with tendency to suicide, attack now in fearfully increasing proportion the working classes and the inhabitants of the country, whereas they appeared formerly to be the special lot of the rich and surfeited class. Lastly, imbecility, congenital or acquired, idiocy, and other more or less complete arrests of development of the faculties of mind or body, indicate in greatly increasing numbers, the existence of individuals who receive in fœtal life the principle of degeneracy.

"At the same time, statisticians reveal to us corresponding facts. The ever increasing number of suicides, of crimes against order and law; the monstrous precocity of young criminals; the degradation (*abâtardissement*) of the race, which, in many localities, can no longer fulfil the conditions required for military service; all these are undeniable facts, and show significantly enough, that the solicitude of European governments has been justly alarmed thereby.

"In presence of a moral and physical aspect so grave as this, I have been led to inquire whether the increasing number of the insane, or the more serious complications of their state, were not connected with the existence of general causes, modifying most seriously the present, and menacing the well-being of future generations.

"The necessary connexion of the causes of degeneration is no longer to me a matter of doubt. I cannot longer separate the study of mental maladies from that of the causes which produce fixed and permanent degenerations, the presence of which, in the midst of the healthy part of the population, is a subject of incessant danger.

"If this be so, the treatment of mental alienation cannot be regarded as independent of all the means which can possibly be made use of, to ameliorate the intellectual, moral, and physical condition of the human race."

With this extended view of the subject, M. Morel proceeds to examine into the various influences of climate, nutrition, social and moral condition, habits, &c., which appear to be exerting a baneful influence upon society in parts or as a whole; indicating the special forms of degeneration due to each individually; and also those which are due to mixed causes. The author's subject is susceptible of the following divisions:—

1. Original unity of the human race.
2. Influences producing natural modifications and morbid degenerations.
3. Degenerations due to poisonous agents—
   *a.* Alcohol. *b.* Opium. *c.* Hachisch. *d.* Tobacco. *e.* Lead.
4. Degenerations due to imperfect nutrition, from
   *a.* Diseased rye. *b.* Diseased maize. *c.* Exclusively vegetable and insufficient diet.
5. Degenerations due to hereditary influence.
6. Degenerations due to the geological arrangements of the soil, as *Cretinism*, &c.
7. Various mixed causes.
8. Curative indications.

What are we to understand by a degeneration of the human race?

Man is not the product of accident; nor yet the last manifestation of imaginary transformations. "Created to attain the end appointed by Infinite Wisdom, he cannot do so, unless the conditions which ensure the permanency and progress of the race, be more powerful than those which tend to destroy and deteriorate it." That there are elements of deterioration and disintegration at work upon humanity and life in general is a very widely spread belief. Bichat says, that such is the mode of existence of living creatures, that everything around them tends to destroy them. This is the expression of an antagonism between living and inert matter, which has formed the foundation for so many philosophical systems; some attributing all evils to unnatural social systems; others to the depravation of the moral sense; and others again to the original corruption of human nature. M. Morel takes no exclusive view in favour of any opinion, but considers the truth to be found in a combination of all.

"Placed in new conditions, the primitive man has experienced the consequences; and his descendants have been able to escape neither from the principle of hereditary transmission, nor from the influence of those causes which, by affecting the health, tended to remove them still further from the primitive type."—"These deviations have produced varieties, of which the one part has constituted races capable of propagating themselves with a persistent special typical character; whilst the other has introduced amongst the races themselves those abnormal conditions, which are to form the subject of our investigations, and which I designate under the name of Degenerations."—"These also have their distinctive characters and types, referrible to the various causes producing them."

"One of the most essential characters of these degenerations is that of hereditary transmission, but under conditions much more grave than those attending ordinary heirdom. Observation shows, that failing certain exceptional elements of regeneration, the offspring of degenerate beings present types of *progressive* degradation. This progression may attain such limits that humanity is only preserved by the *very excess of the evil,* and the reason is plain; the existence of degenerate beings is necessarily bounded; and it is not even necessary that they should reach the *last degree* of degeneracy in order to be smitten by sterility, and become incapable of transmitting the type of their degradation."

*Varieties of the Human Race, and Formation of Degenerations.*—"The origin of the first deviations from the primitive type, was connected with the original necessity for man to harmonize external nature with the laws of his own preservation. This strife still everywhere continues; and man only exists on condition of constantly combating noxious influences, and all the hurtful elements amidst which circumstances may have placed him."

Buffon says that three causes tend to produce changes in animal constitution—climate, nourishment, and domesticity. Allied as man is *physically* to other organized beings, he must necessarily be submitted to the same influences under certain limitations; but to attain just ideas we must in his case substitute for domesticity, the aggregate of manners, customs, education, civilization, and the like.

To these influences are due the modifications of type known to naturalists as the white, black, yellow, red, and brown races of men, all originally proceeding from one stock or species. (Buffon.)

The one great fact adduced in proof of this position is, that all the varieties can unite to propagate the race; and their progeny, however apparently dissimilar the parents, are fertile, and can continue the species. But this is only so far as regards the *natural* modifications of, not the *morbid* deviations from, the primitive type.

"The more profound is the degeneration, the more difficult does it become to realise this great fact of the possibility of transmitting the race. *Morbidly degenerate beings cannot form a race.* The continuity of a morbid variety (*variété maladive*), such as that of cretins, depends on the union of the sound part of the population with those more or less profoundly affected with the poisonous influence."

The unity of race is no less important for the classification of disease than for the stability of the science of anthropology. Some direct analogical evidence as to modifications of primitive type is therefore brought forward to indicate the operations of the three great causes alluded to by Buffon, and the manner in which constitution and temperament are modified by circumstances, and even so as to produce disease.

*Modifications in the Organizations and Instincts of Animals.*—When animals are transported into a new climate, not only the individuals but the race require acclimatisation.

"Nothing is more curious than the successive changes produced in animals by domesticity and their return to savage life. Reduced to captivity, they not only lose many of their natural instincts and acquire new ones, but remarkable physiological transformations occur. M. Roulin relates in connexion with the introduction of pigs into Saint Domingo, that many of them escaped and became wild; and it is remarked that their ears have become straight again; their heads have become widened and elevated behind; and the colour, instead of those varieties met with in the domesticated state, is almost uniformly black. The same has been observed in other countries, where the pig returning to the wild state, has become in form, colour, and texture of hair like the wild boar. A very important fact, in its physiological and hereditary bearing, is noticed with regard to the lactation of cows. The constant practice of milking these creatures during many generations, has caused the secretion of milk to become a constant function in the economy. In Colombia the abundance of cattle and sundry other circumstances have interrupted this habit of secretion; and in a very few generations the mammæ have returned to the normal small size. Certain habits of progression are also hereditary, as the mode of walking of the Naragganset horse. In other cases instincts are developed and become hereditary through habit, as in the dogs that are brought up to hunt the peccari. Their young ones know instinctively how to attack this ferocious brute, whilst the offspring of untrained dogs are devoured in an instant. Barking appears also to be an acquired but hereditary habit. Wild dogs do not bark, but howl. The young of domesticated dogs bark even when removed from their parents early; but dogs which become wild after being domesticated lose the habit of barking, and howl again. The same is observed in cats."

Many other instances are given, but these are sufficient to illustrate the point in question, and to justify the deduction, that man himself is not unamenable to the powerful influence of physical agencies; seeing that he is a being composed of the same materials, and constructed on the same principles, as those over which he has dominion. Doubtless in the constant strife with the elements to adapt them to his constitution, the latter is modified in some degree, and thereby adapted to the particular circumstances under which he is placed. This within certain limits cannot be considered morbid, nor a degeneration. Hence arises another question, treated in the next section.

*On the Difference between Natural Modifications which produce Varieties, and Abnormal or Morbid Changes, which produce Degenerations.*—In the strife above mentioned, the constitution may be modified just sufficiently to adapt it to surrounding nature; but an exaggeration of these causes may pass on to what becomes degeneration. It is not always easy to trace the line of demarcation, but certain instances are here given in illustration. There are amazing differences between the Esquimaux who gorges himself with whale's blubber, and that "African starveling" who pursues the lion under a tropical sun; between the fisherman of the North, covered with seal-skin, and the hunter of the Sahara; between the luxurious Eastern and the energetic European. But these are all natural modifications to suit climate. The following is also an interesting example, quoted by M. Morel from M. D'Orbigny. It refers to the Incas or Quichuas, the type of whose conformation is very accurately drawn, and closely approximates to that of the Mexicans. It is unnecessary to enter upon more than one point. Notwithstanding their very short stature, they are represented as having more massive forms than other tribes :—

"The Quichuas have very large square shoulders, and the chest is excessively voluminous, being arched and very long, so as to increase greatly the size of the trunk. In the women it is the same, and the throat is always large. . . . . This is a striking organic fact; and is explicable on the principles of adaptation already mentioned. The plateaus inhabited by this race are always included between the limits of 2500 and 5000 metres of elevation (2750 to 5500 yards) above the level of the sea. At this altitude the air is so much rarefied, that it is necessary to take a much greater volume of it into the chest, in order to provide the system with a due amount of oxygen. During infancy, therefore, and the whole period of growth, the chest is developed irrespective of the growth of other parts. Confirming this theory, is the fact that the lungs themselves are altered in texture; the cells are enlarged, and, in consequence, the whole volume of the lungs is increased."

The next instance brought forward is one in which we find man in the act of undergoing an actual organic change. "When men of the North," writes Dr. Buchez, "emigrate to the Torrid zone, changes take place well worthy of attention. The general circulation is excited, the blood is diminished in quantity, and the arteries are less full. The circulation of the *vena porta* is increased, and a superfluity of bile is produced; the liver becomes enormous, and appears to supplement the respiratory function, as in fœtal life. The muscular force has less energy."

Both these instances are only illustrations of the natural modifications of structure and function, intended to adapt the constitution to exceptional circumstances. But these changes, similar in nature, may go on to excess, and become morbid. M. Melier says :—

"Visiting the village of Hiers, we saw children of twelve years old who appeared but six or eight, so puny and undeveloped were they. Their tint is not merely pale, but tarnished, and of a dirty grey; at once meagre in limb, and swelled in feature, they have only the belly developed; and they have almost all incurable congestions. For some time the canton (a marshy district) could not furnish the military contingent. It often happened that of all the men drawn for service, not one was found fit; sometimes none were found of age to be recruited—all had died, the most part in their infancy."—(*Rapport sur les marais salants.*)

"In studying the action of the constitution of the soil upon man, we shall arrive at a point of degradation in which, according to some naturalists, he no longer suggests the idea of his species. He is not only imperfect but degenerate. Those who can still contribute to reproduce their kind, do it under condition of an ever downward progression. The more advanced are impotent; they present the type of *cretinous degeneration* in its extreme manifestation."*

The remainder of the section is occupied by notices of the lowest forms of *natural* modification, such as the Hottentots, Bosjesmans, &c., as distinguished from the true morbid degenerations. The general conclusions are these :—

"We conclude then, that the intellectual inferiority found in certain races, does not necessarily involve the idea of a *morbid* state, as observed in true *degeneration.*

"Climatic influences have induced certain changes, certain typical characters, transmitted from one generation to another, and so produced the *varieties* of race. These varieties can mix with superior varieties, and, under favourable circumstances, can thereby ascend towards the more perfect type.

"The same is the case with intellectual manifestations. Their inferiority in this respect is not sufficiently general and permanent to permit the idea of distinct species. The intelligence appears merely dormant, and to be susceptible of cultivation up to the normal point of the race. The intellectual inferiority due to morbid degeneration of type is so distinct from that just noticed, that we are justified in adopting the following conclusions :—

"Between the intellectual condition of the lowest Bosjesman and the most civilized European, there is much less difference than between the same European and this degenerate being, in whom arrest of development is due to cerebral atrophy, congenital or acquired; or to any other cause inducing the morbid state which we designate by the name of idiocy, imbecility, or dementia.

"The first is susceptible of *radical amendment*, and his progeny may ascend to a higher, or even the highest type. The second is only susceptible of *relative* amelioration, and hereditary influence will *always* weigh upon his descendants. He will remain all his life what he is in reality, a specimen of degeneration in the human race, an example of *morbid deviation from the normal type of humanity.*"

The causes of degeneracy may be briefly summed up as follows :

1. *Degenerations by Poisonous Influences.*—These include marsh miasmata, and all the geological and climatic influences which tend to the production of hereditary cachexia; chronic poisoning by alcohol, by opium, tobacco, and various other narcotics.

2. Humanity is periodically condemned to certain scourges, which bring in their train fatal modifications of organism. Of these are famines and epidemics of various kinds, generally attended by, or consequent upon, extraordinary perturbations in the regular order of the seasons, and in natural phenomena.

3. Insufficient, bad, or exclusive nutriment impoverishes the constitution, and tends to the degeneration of the species. These

* M. Morel, p. 34.

produce certain special affections of an eminently deteriorating character, as Pellagra.

4. Another source of degeneration is the social medium in which man is placed.

"It is not enough for man to have conquered external nature; he must also strive with his internal nature, or rather the factitious nature imposed upon him by the social condition in which his existence is passed. The practice of dangerous or unhealthy professions; the habitation of situations crowded or insalubrious, expose the organism to new causes of decay, and consequently degeneration. In spite of the progress of science, it is impossible that he should not be modified by the evil conditions of a life devoted to certain manufactures, and the use of certain toxicant agents, or by the necessity of passing much of his life underground. Add to these general conditions the profoundly demoralizing tendency of misery, lack of instruction, failure of prevision, abuse of alcoholic liquors and venereal indulgences, and the insufficiency of nutriment; and we shall form an idea of the complex circumstances which tend to modify the temperaments of the lower class."

5. *Of the Degenerations which result from Infirmities either congenital, or acquired in Infancy*, M. Morel observes :—

"The child may be born with a brain incapable of fulfilling its functions, because it is atrophied or altered in its intimate texture, or because its bony case is formed so as to prevent its due development. Then, those functions of the organism over which the nervous system presides are performed in a vitiated manner. The child remains *degenerate*, because the instrument which is indispensable to the exercise of the human faculties only performs its functions imperfectly, or morbidly. He is affected not only as to the development of his intelligence, but as to that of the organism. . . . The child may suffer from hereditary disease, receiving, whilst in the womb, the seeds of degeneracy; or, without this, he may be exposed to convulsive or tuberculous affections early in life, which lead to the same consequences as congenital imbecility, or idiocy; he may also be subject to certain practices arising from ignorance, superstition, or other motives, such as compressing the head, to give it a form in accordance with certain singular ideas of typical beauty."

6. Blindness, and the deaf-mute condition are included in this brief enumeration of the causes of degeneracy, not as being so serious in themselves as some of the others, but because, in default of proper education, creatures so affected are certainly imperfect beings, and are likely to transmit some forms of imperfection to their offspring; though not necessarily their own deficiency. The fact of the existence of one hundred thousand persons so affected in France, is brought forward as a powerful reason for including some means of extended care over these unfortunate beings, in any system of general hygiene.

In the preliminary notice of degenerations in relation to hereditary influences, there are some observations too important to be passed over.

"We do not fear to avow that the principal interest which will attach to these considerations will arise from an exposition of the errors into which we have sometimes fallen in reference to certain forms of mental disease. Far be it from us to discourage those who are animated by the desire to do good to their kind; but we believe it useful to forewarn them against hopes most cruelly to be disappointed, if they do not keep in view, that hereditary tendency is not an isolated fact; and that the incurability against which often our best efforts are shattered, is but the fatal termination of a series of anterior existences, which are morbidly summed up and represented in one doomed individual. It is in the treatment of mental alienation that we have been exposed to the greatest deceptions. We have ventured to predict recovery, not having this aspect of matters in view, in acute cases; but calm has superseded the general disorder, and we have had to recognise that the individual had ceased to live intellectually. Numerous facts have proved to us that the incurability of these cases is not so much in relation to such or such a *form* of disease, but to certain hereditary influences, the study of which permits us to draw the following conclusions:—

1. There exist certain individuals who resume in their own person the morbid organic tendencies of many previous generations.

2. A development of certain faculties, sufficiently remarkable, may occasionally throw a more hopeful light upon the future of these individuals; but *their intellectual existence is circumscribed within certain limits which they cannot pass.*

3. The conditions of degeneration in such individuals reveal themselves not only by typical exterior characters, as smallness or malformation of the head, predominance of morbid temperament, anomalies in the structure of organs, special deformities, impotence, and the like; but also by the most strange *aberrations in the exercise of the intellectual faculties and the moral sentiments.*

Some general observations upon classification, and upon the principles involved in prophylaxis and hygiene, conclude the prolegomena. The following remarks are so forcible that we give them entire:—

"What are asylums but the concentration of the principal degenerations of the race? Because one is placed here as a maniac, an epileptic, an imbecile, or an idiot, he is not the less—in the majority of cases, if not all—the product of one or more of the causes of degeneration now enumerated. We, as physicians, better than others, are able to appreciate the influence of alcoholic excesses—of hereditary affections—of misery and privations—of insalubrious professions—of unhealthy localities. If, then, the causes of so much evil may yield before the efforts of the administrative authority, surely we are right to appeal to it. The influence which we can exert in our own department is undoubtedly great, but still small when confronted with the great mass of incurable cases committed to our care. We must not, then, remain inactive contemplators of so many destructive agencies. Medicine alone can sufficiently appreciate the causes producing degeneracy of race; to it alone, therefore, it belongs to point out the positive indication of the remedies to be employed. I admit that the experience to be acquired in even a long career, scarcely would suffice to resolve a few of the problems proposed in this book; but I say, with the author of the *Introduction to the Science of History* (M. Buchez)—'No one knows when his hour may come—no one knows if the idea which he bears may die with him. In this uncertainty only one part remains—to make haste, that when the night comes, our work may be done.' "

*On Degenerations Caused by Poisonous Agents.—On Chronic Alcoholism.*—M. Magnus Huss calls the disastrous effects produced by the abuse of alcoholic liquors by the name of Chronic Alcoholism. Entering the system in large quantities, it modifies fatally the constituent elements of the blood, and acts as a poison. The symptoms of this poisoning are those of alternate excitement and depression. Partial paralyses are but the precursors of more grave affections, which terminate finally in *general paralysis, deterioration* and *ultimate loss of intelligence.* The cadaveric lesions are correspondingly serious. But this is not the worst. The physical degradation, the complete perversion of the intelligence and the moral sentiments, do not remain isolated facts, terminating with the individual. There is no malady in which hereditary influence is so marked and characteristic. "If congenital imbecility and idiocy are the extreme terms of alcoholic degeneration, many intermediate states reveal themselves to the observer, by aberrations of intelligence and perversions of the moral sentiments, so extraordinary as to be unaccountable on the mere theory of warping of the moral nature. . . . It would be impossible, rejecting the data of hereditary influence, to account justly for many moral and physical monstrosities. Perhaps, placing ourselves in the scientific point of view of the question, we may be able to cast a new light upon intellectual conditions hitherto inexplicable, and to render a great service to legal medicine, to education, and even to morality, by assigning to the sad victims (personal and hereditary) of alcoholism, their true place amongst degenerate types."

After entering somewhat extensively into the history of alcohol, M. Morel proceeds to describe the progressive symptoms of alcoholic poisoning. A case is detailed at great length, in which the following symptoms occurred progressively:—First, after ten or twelve years' abuse of drinks, came on repeated attacks of delirium tremens; then habitually trembling hands; disordered sensations, such as occasional blindness; trembling tongue, troubled sleep, disgust for all food; formication and subsultus; trembling legs and advancing paralysis; then partial anæsthesia, becoming complete in the fingers, toes, and inner part of the thighs; then vertigos and serious hallucinations. At this period a strenuous effort was made to stop the downward course, and for a little time successfully. Again the evil courses were resumed, and again the old train of symptoms occurred, with emaciation, and frightful cramps and spasms. Again a cessation of drinking, and again a relapse. The final condition is thus described :—

"Arrived at this sad period, there was no longer hope of amendment. Deprived of intelligence, lost to all moral sense, his strength diminished from day to day; and nothing could now arrest the progressive and fatal march of the symptoms. The skin became like parchment, the legs were œdematous, and the digestion profoundly troubled. The delirium, though continuous, had now no violent exacerbations. He muttered unintelligibly, his look was stupid and haggard, his appearance brutal; and when death came to terminate this sad existence, consciousness had long ceased. The paralysis was general, and this deplorable victim of alcoholism had fallen into the most hideous state of degradation."

Alcohol, then, produces a malady presenting the symptoms of true poisoning, and one of a specific character. The only disease likely to be confounded with it is that known as "general paralysis." The symptoms in general are trembling of the feet and hands, diminution of strength, paralysis, subsultus, cramps, and spasms. It is only in an after stage of the disease that convulsions and epilepsy occur.

In the nervous system we notice at first formications, exaggeration of sensibility, and neuralgic affections; later, diminution of sensibility, perversions of the senses, and difficulty of speaking.

The circumstances attendant upon the generative function are peculiar. There is at first an exaltation, followed by depression, and finally extinction or impotency. Dr. Huss thinks that the same applies to women. It follows from this that, in general, hereditary influence is only operative during the earlier periods, when the function appears to receive an accession of vigour. Yet a precocious impotence seems to attack the succeeding generations; and thus, by a preservative law of nature, the degenerate race becomes speedily extinct. The offspring are not only afflicted with congenital intellectual feebleness; but along with this intellectual and moral degradation comes the impossibility of perpetuating the race, notwithstanding the normal development of the generative organs.

In the intellectual sphere of the nervous system the symptoms coincide with those of the physical order. At first, a redoubled activity in the evolution of ideas; next, alternations of excitement and depression; finally, stupor. The acute symptoms, as shown in delirium tremens, are sufficiently familiar.

The symptoms due to other functions are—vomiting, furred tongue, diarrhœa, and flux. The liver is much disordered, and autopsy often reveals cirrhosis and atrophy. The kidneys are often affected with granular disease. The heart is excited to overaction, and hypertrophy is often the result, followed not unfrequently by fatty degeneration. There is also an alteration in the elements of the blood, which appears to abound in fat The arteries are strikingly enlarged in calibre. (Huss.)

The most serious pathological lesions are rupture of vessels and extravasations of blood, producing apoplexy; atrophy, general or partial, of the brain—the former most common; serous effusions on the surface of the brain, or in the ventricles; opacity and thickening of the membranes, and adherence to each other and to the cranium.

The diagnosis between alcoholism and general paralysis is worthy of attention. In each there is a peculiar trembling of the hands, weakness of the lower extremities, feebleness of speech, &c. In alcoholism, these symptoms are said by Dr. Huss to cease when the cause is given up; and, even without that, there are occasional diurnal remissions never observed in the simple progressive paralysis. In this latter, the affections of the sight so common in the former are not observed, nor the characteristic formications in the extremities. The phenomena of digestion afford a very striking distinction. In alcoholism they are always much disturbed; in paralysis the appetite is normal, or increased to voracity. There are likewise differences in the mode of invasion of the insensibility, and in the nature of the hallucinations and delirium.

"Whether general paralysis, which terminates the life of so large a proportion of the insane, be *always* induced by excess of alcoholic drinks, it is not necessary here to examine; or if these excesses have contributed their quota of destructive activity to the already existing nervous malady; but it is certain that our asylums contain always a large proportion of cases having no other traceable origin. Out of 1000 cases, the details of which I have collected, there were 200 in which no other cause existed." (M. Morel, p. 109.)

There are different varieties of alcoholism observed in our asylums, according as it is induced directly in the individual, or inherited from the parents. In the latter case, the victims have come to terminate their days in the last convulsions of general paralysis, and in a state of the most profound moral and physical degradation. The former class, *removed earlier from their evi*

*courses*, pass an existence but little more enviable ; of which dementia, stupor, the absence of intellectual vigour, and the abolition of *all moral sentiment*, form the prominent characters. This class is very numerous. There is no special delirium ; their existence is automatic ; the only wish expressed is to escape, and resume their vicious excesses. General paralysis is not ordinarily the termination of this class of cases—they present many of the characters of alcoholic poisoning, stopping short of that series of progressive lesions which terminate in general paralysis. The usual termination is complete marasmus, with general or partial dropsy, and irrestrainable diarrhœas.

"Thus are established two distinct forms of alcoholic degeneration —one, in which the victims have passed through a determinate series of nervous lesions, both of a physical and intellectual order, to general paralysis ; the other, where they remain stationary, and drag on a weary existence, characterized physically by cachexia and marasmus, and morally by the manifestation of the most depraved tendencies and the most profound degradation.

"We have now to study two other classes—(1), those whose malady has been developed under direct hereditary influence; and (2), those whose depraved addiction to drink may be attributed to special affections of the organism."

In the first, or hereditary class, there are also varieties. The children may simply inherit the tendencies of the parent; but what was habit in the one, becomes an instinct, perhaps uncontrollable, in the other; and the termination is as already described.

But it is not necessary that the descendants of such parents should commit the same excesses, in order to present the type of progressive degradation. Some are born *completely degenerate*, that is imbeciles or idiots; others live intellectually up to a certain age, beyond which they stop, and fall into a state resembling dementia.

"After painfully attaining a certain status—after having laboriously acquired a profession—they find themselves incapable of further progress, and begin to retrograde. They experience *critical phases*, which fix the conditions of their future existence; for instance, the occurrence of puberty, of incidental maladies of a physical or moral nature, and the like. In these cases, sudden and irremediable transition to idiocy is the fatal termination which awaits them."—(p. 115.)

Several cases of deep interest are given in illustration of this latter phase, but they are too extended for quotation. They tend to indicate the almost utter impossibility of escape from the hereditary type once stamped upon the race; and the futility of placing any dependence upon the most solemn and reiterated vows of amendment from those who are once subjected to this influence. M. Morel appends to these instances a remark, part of which we shall give in his own words :—

"When patients of this class have passed some time in a house of recovery, they return apparently to better sentiments, and make the most solemn promises of amendment. The intervention of authority. and family requirements, then force us to consent to their liberation, of which *all* have ultimately cause to repent.

"JE N'AI JAMAIS VU GUERIR *les malades dont les tendences alcooliques avaient leur point de départ dans les predispositions héréditaires leguées par les parents.*

"Their exit from the establishment was at once followed by a repetition of the same acts. It was necessary to isolate them again ; and each time there was an advance of degradation."—(p. 118.)

One case is too important not to notice briefly, as it seems to resume in itself all the sad train of phenomena involved in these considerations. The great-grandfather of the young man in question indulged in drink, till it became veritable dypsomania. He was killed in a pot-house quarrel. His son, the grandfather, followed in his footsteps, was brought a maniac to the asylum, and died ultimately of general paralysis. His son, the father, was of comparatively sober habits, but *not the less* did the hereditary taint show itself ; he became insane on the idea of persecutions, &c. His son, the young man in question, was brought to the asylum at the age of eighteen, attacked eight months before without ostensible cause, by mania, the transition to complete idiocy. Thus we see—

"In the 1st generation.—Immorality, depravity, alcoholic excess, brutish disposition.

"In the 2nd.—Hereditary drunkenness, maniacal accessions, and general paralysis.

"In the 3rd. — Sobriety, hypochondriac and lypomaniacal tendencies ; systematic ideas of persecutions, and homicidal impulses.

"In the 4th.—Weak intelligence originally, access of mania ; stupor ; transition to idiocy ; *finally, extinction of the race.*"

It remains to make a few observations upon the tendency to alcoholic drinks, resulting from certain pathological changes. M. Esquirol long ago showed that, whereas the abuse of fermented liquors is often the result of degradation of mind, vices of education, and evil example ; there is sometimes a morbid irresistible impulse which drives certain individuals to such abuse. He has noticed this impulse at the cessation of menstruation, and in the case of an advocate, suffering from a cutaneous affection. M. Morel has also observed a case similar to the latter. Sometimes the tendency is observed to be irresistible at the period of menstruation, and during pregnancy. Out of 200 cases of alcoholism M. Morel attributes 35 to organic disease. General paralysis was the cause in 10 cases, and organic disease of the heart in 3. In 6 hypochondriacs, and 4 hysterical women, the most marked tendency to alcohol complicated ultimately the original affection. In 16 it appeared due to hereditary tendency to some form of disease *not* alcoholism. In these latter cases the disposition to *steal* was very prominent; they seem to be amongst the most incurable of all mental alienations.

Thus, there are four fatal forms of alcoholism :—

1. Those who have gone through every form of alcoholic poisoning, and terminate their career in general paralysis and dementia.

2. Those who at an earlier period of their vicious career have been secluded in the asylum, and live as above related.

3. The descendants of the two previous classes, including born idiots and imbeciles, and those who live intellectually up to a few years of age, and fall into dementia.

4. Those who are led to alcoholic excess by previous disease or predisposition.

M. Morel concludes his extended notice of alcoholic poisoning, by remarking upon the constant increase, throughout Europe, of this particular cause of degeneration. Its effects in causing degeneration of *race* are traced in another part of the work; the observations, so far, having chiefly applied to individual deterioration.

*On Degenerations resulting from various Vegetable and Mineral Poisons.*—This subject divides itself naturally into two departments: (1) the effect produced upon the animal economy by certain narcotics, of which the Orientals make use (and after them, others) to produce factitious excitement, in default of spirituous liquors; and (2) the poisonous action resulting from the employment of certain mineral agents in commerce, manufactures, and the arts, as lead and mercury.

M. Morel enters into the history of the use of intoxicant plants as excitants, showing their employment from the earliest times of which we have definite records, and their almost universal use now. He sketches briefly the *kava* of the Polynesians, the *niopo* of the Ottomaques; the betel nut, the *kaad*, the nuts of *kola* and *coca* of the Chinese, Ceylonese, &c., all used for one and the same purpose of intoxication or stupefaction; and then passes on to the three most universally spread narcotics, Hachisch, Opium, and Tobacco.

1. HACHISCH.—The Indian hemp *(Cannabis Indica)* forms the basis of most of the intoxicating preparations used in Egypt, Syria, and most Oriental countries. The leaves are smoked alone or mixed with tobacco. But the most celebrated preparation is the fatty extract known as hachisch, which seems to be butter charged with the active principle. It is too nauseous to take alone, but is made up into various forms of cakes and electuaries, sometimes mixed with aphrodisiacs, and sometimes with other narcotics, as opium, stramonium, &c. The effects on the system have been so often described, and are so analogous, in many respects, to those of opium,* shortly to be mentioned, that it is not necessary to recapitulate them. They are all referrible to the nervous system. The final results are thus alluded to by M. Moreau :—

"Besides the habitual hallucinations which the extract of Indian hemp produces in some individuals, I think its prolonged usage induces incurable dementia. I have reason to believe that such is the case in many persons met with in the cities of Egypt, who are venerated as holy men (*santons*) by the people, but who are merely fallen into a state of dementia from the use of hachisch."

2. Opium.—"At no period of time has humanity witnessed a fact like that we have now to consider," says M. Morel. "Three hundred millions of individuals, united under one absolute government, speaking the same language, and having identical religious notions, present to us the sad spectacle of a people menaced, as to its dearest interests, by the most fatal and degrading habit that it is possible to conceive—that of smoking opium."

An idea may be formed of the frightful increase of the consumption of opium in China by the following figures. China is selected as a typical illustration of the effects of this practice on the race. In 1810, 2500 cases of opium were sent to Canton ; in 1820, 4770 cases; in 1830, 18,760 cases; and in 1838, 48,000 cases! And this in spite of the laws enacted against it! laws which the lawgivers are the first to infringe and set at nought.

The effects of smoking it, immediate and remote, are thus described :—

"The first impression is a feeling of content and slight excitement, manifested by loquacity and involuntary laughter. Sometimes there are fits of anger. Soon the eyes become brilliant, and the respiration and circulation are quickened and excited. At this stage of the nervous exaltation the smoker feels a peculiar comfort (*un bien-être tout à fait particulier*), and the temperature is augmented. The impressions are lively, and the imagination wanders into strange illusions. Now we observe a phenomenon frequently remarked in mental alienation. Facts and ideas, long forgotten, present themselves to the mind in all their original freshness. The future appears all bright, and every happiness ever wished for appears realized by the smoker. If he continues smoking, exaltation gives place to depression and utter prostration.

* For the differences of the phenomena produced by these two agents, consult Pereira's "Materia Medica," pp. 1238, *et seq.*

The action of the senses is suspended. He hears nothing; he becomes silent; his face becomes pale, his tongue hangs out; a cold sweat inundates the whole body; and insensibility supervenes, often lasting for several hours. The awakening is what might be expected after such a debauch."

Such are the immediate effects, but neither tobacco nor Indian hemp (nor *perhaps* alcohol) are to compare with opium either in the constitutional results, or in the difficulty of breaking the habit. Except some few smokers, who, thanks to an exceptional organization, can restrain themselves within the bounds of moderation, all the others attain rapidly a fatal termination, having passed in quick succession the stages of idleness, debauch, misery, the ruin of their physical strength, and the utter depravation of their moral and intellectual faculties. Nothing can cure an advanced smoker of opium.—(Huc.) " C'est une atonie dégoûtante, une prostration absolue de toutes les facultés et de toutes les energies."

" But the action of opium is more pernicious than that of alcohol in another particular—viz., the *rapidity* with which the nervous lesions declare themselves. Given the period at which a person begins to smoke opium, it is easy to predict the time of his death; his days are numbered. The physiological effects are uniform, and succeed each other with an unvarying regularity.

" According to Dr. Ainsley, a considerable fattening first occurs; then failure of strength, and irregularity of walk; then the memory is lost, the intellectual faculties fail, and dementia results. The termination is similar to that of the victims of alcoholism in Europe. . . . . *No smoker of opium attains an advanced age*, and their offspring are blanched, miserable, and struck with premature mental decay. For obvious reasons, we have not yet the same opportunity of tracing the ultimate degenerating effect of this practice on the race, as we have of that of alcoholism; but it cannot be doubted that the same law will hold good; and we cannot but be alarmed for the intellectual, physical, and moral future reserved for China, Sumatra, and the other countries where this practice obtains."

M. Morel concludes this notice of opium by a question especially interesting to this country:—

" Is it true, as some authors affirm, that the habit of smoking opium has invaded the capital of England? If it be so, it is impossible to calculate the evil impending. Meanwhile, statistics point to a sad conclusion. In 1830, there were 103,718 pounds of opium received in London, and in 1852, 250,790 pounds!"

3. TOBACCO.—M. Morel expresses himself very guardedly when speaking of the effects of tobacco. He thus introduces the subject:—

" What may be the part which tobacco plays in the production of degeneration? And admitting even that its degenerative action is an ascertained fact, how far would it be good medical hygiene to attack the usage of tobacco, which has become for all nations not only a habit, but an imperious necessity, to be satisfied at any risk? . . . . I have no intention of attacking its use, and this for many motives; first, it is far from being proved that the habit of smoking *in moderation* is in any way injurious; and, secondly, it would not be without danger to invoke the force of an absolute legislation against a habit passed into such an irresistible necessity."

M. Morel seems to think that a large proportion of men *will have* either tobacco or opium, and of the two evils he prefers the former. He considers that there are certain questions still *sub judice*, leaving out of question the moderate use of tobacco—viz., the results of its abuse; the effects upon the system of those engaged in the manufacture of snuff and tobacco; the influence of its special culture upon the internal economy of a country; and the effect of its introduction upon the manners and social customs of a nation.

In answering the question—"Is tobacco injurious to the health?" M. Morel takes simply the fact that nicotine is a virulent poison, and that it is unlikely that such can be introduced into the system in large and repeated quantities without injury. He relates the result of its application to wounds in animals, and its introduction into the stomach, &c., those experiments which are so well known, and which a recent and still continuing controversy has made so familiar to us. He then adds:—

"Were we then to judge *a priori* of the evil consequences to be anticipated from the use of tobacco, we might well be terrified at the prospect. But observing facts and results, we are compelled to conclude that the dose of nicotine absorbed must be too small to produce such serious results except in a small number of cases. . . . . We may shortly sum up the evils quoted by authors. The first attempts at smoking produce nausea and vomiting, but the economy soon habituates itself to the practice. It is injurious to adults who have not reached their development; much more to children. The great quantity of saliva secreted, interferes seriously with the functions. Young smokers are generally pale and meagre, and the phenomena of nutrition are imperfect. There is alternate excitement and depression of the nervous system; and it is said that inflammations of the throat and respiratory passages are common. Add to this, that the smoker generally drinks, and passes much of his time in a vitiated atmosphere, and we shall not be astonished at the sad prognostications of many authors."

After alluding to certain cases of clearly defined nervous lesions *following* the immoderate use of tobacco, M. Morel passes on to discuss the effects of the manufacture of this substance on the workpeople; and though he *believes* that these processes cannot be accomplished without producing some deleterious effects, yet again consulting facts, he feels "bound to suspend his verdict." M. Melier thinks more unfavourably; from his observations he believes that a change takes place in the blood of those thus employed, which amounts to a kind of poisoning. This is in accordance with some views recently propounded in the controversy alluded to.

The subject of tobacco has been lately brought very prominently before the profession and the public, by some observations made by Mr. Solly at St. Thomas's Hospital; and the remarks, excellent in themselves, are much more so as proceeding from so thoughtful and experienced a source. They cannot be too much nor too carefully considered. In relating a case of paralysis, and speaking of its causes, he says:—

"There was another habit also in which my patient indulged, and which I cannot but regard as the curse of the present age—I mean smoking. . . . . I know of no *single* vice which does so much harm. It is a snare and a delusion. It soothes the excited nervous system at the time, to render it more irritable and more feeble ultimately. . . . *I believe that cases of general paralysis are more frequent in England than they used to be; and I suspect that smoking tobacco is one of the causes of that increase.*"

On another occasion he writes:—

"I believe if the habit of smoking advances in England as it has done for the last ten years, that the English character will lose that combination of energy and solidity that has hitherto distinguished it, and that England will sink in the scale of nations."

It is unnecessary further to quote from the interesting letters of Mr. Solly on this subject; they are in the hands of every one.* An animated correspondence has arisen, which it is to be hoped will result in some accurate and specific observations on the influence of tobacco in the production of disease. Meanwhile many interesting facts are placed on record. A few of the writers seem inclined to trace almost all social and physical evils to this practice, whilst others consider it as innocuous. It is reprobrated because it produces insanity, paralysis, consumption, laryngitis, tonsillitis, short sight, emaciation, dyspepsia, and an infinity of minor disorders. It is upheld because it is pleasant; because it is a valuable therapeutic and hygienic agent, a preservative against cold and starvation, a substitute for food, a solace to the weary, whether of mind or body. One writer attempts to settle its value by an appeal to final causes, asking—"Why was tobacco created, if not to be smoked?" perhaps overlooking the fact that the same trenchant argument applies to every known vice.

* *Vide* "Lancet," Feb. 14th, 1857, &c.

Amid all this gleams of valuable information appear. Dr. Pidduck, writing about smokers, as seen at the Dispensary in St. Giles's, says:—

"Leeches were killed instantly by the blood of the smokers, so suddenly that they dropped off dead immediately they were applied. . . . Fleas and bugs rarely, if ever, attacked the smoking parent."

And what is very important in reference to the subject now under treatment:—

"If the evil ended with the individual, who, by the indulgence of a pernicious custom, injures his own health, and impairs his faculties of mind and body, he might be left to his enjoyment, his *fool's paradise* unmolested. This, however, is not the case. *In no instance is the sin of the father more strikingly visited upon the children than the sin of tobacco smoking.* The enervation, the hypochondriasis, the hysteria, the insanity, the dwarfish deformities, the consumption, the suffering lives and early deaths of the children of inveterate smokers, bear ample testimony to the feebleness and unsoundness of the constitution transmitted by this pernicious habit."—*Lancet*, Feb. 14.

The use of tobacco *in moderation*, and under certain circumstances of great hardship and privation, is upheld by many men of high scientific attainments and sound judgment, as not only not injurious, but beneficial both hygienically, therapeutically, and psychically.

Medical men engaged in the investigation and treatment of the diseases of the brain and disorders of the mind, occasionally have brought under their notice cases of severe nervous disorder and mental impairment, clearly traceable to an excessive and immoderate use of tobacco. Shattered nervous system—premature loss of mental vigour—impaired memory—mental alienation, are too often the well-defined result of excessive tobacco smoking. These are facts that cannot be ignored when considering the question at issue.

If society were in a more natural condition, or one more in accordance with the most obvious rules of hygiene, it is highly probable that no poisonous agent, whether narcotic or stimulant, would be habitually desirable or allowable. It would not be easy to define accurately what *is* "a natural state of society;" but it is easy to say what is *not*. For instance, it is not *natural* for man to pass his life underground, as in the coal mines of this country, and still more in the salt mines abroad; to be exposed, in addition to the ordinary atmospheric vicissitudes, to those of moisture and cold in connexion with sieges, and migrations from a temperate, to either an arctic or a tropical climate; to be immersed perpetually in poisonous or irritating vapours, as in various branches of art or industry; to be suffering the extremes of misery, privation, and hereditary disease. Nor does it appear a *natural*

course of proceeding, that a man should pass his days in the wasting exercise of an arduous and anxious profession; and perhaps, his nights in noting down the results thereof for the benefit of future generations. These and many other conditions suggest themselves at once as a part of those evil influences noticed in an early part of this paper, with which man has to wage perpetual warfare; and it does not appear improbable, that *within moderation*, the use of tobacco *may have* as beneficial an effect in enabling him to resist successfully some of these influences, as any other prophylactic agency may have in other cases. It may be injurious to the *normal constitution normally treated*, but may it not resist or avert the abnormal consequences of a different condition?

So far as to the *use*. The abuse entails certain undeniable consequences. First, dyspepsia and anorexia, with their natural results of cachæmia and partial marasmus. Then ensue those *special nervous lesions*, the nature of which is so clearly indicated by the experiments upon animal life. And finally appear those degenerating effects upon the offspring, which have been before noticed. It must be mentioned in conclusion, and as bearing upon the continuance of the species, that tobacco is supposed to be a powerful anaphrodisiac.

4. *Lead.*—M. Morel gives a case illustrative of lead colic, and partial lead paralysis, and then points out the analogies between this and alcoholic poisoning. There is, in the commencement, trembling, weakness, and paralysis of the lower extremities, and diminution of the general sensibility. Soon there are twitchings and cramps, dizziness, fantastic dreams and hallucinations; and these are exactly the symptoms of the anæsthetic form of alcoholic poisoning. One symptom, however, is wanting in the lead poisoning,—that of formication.

M. Morel adds, referring to the general train of symptoms:—

> "These are the symptoms inseparable from all chronic poisoning; and more than that, they are the essential signs, which announce by their duration and their constant progress, that the individual is smitten in the most important functions, and is tending to degenerative transformation more and more radical."

M. Morel notices another distinction between alcoholic and lead poisoning; that whereas, in the former a toleration of the poison to an enormous amount is acquired, there is none such observable in lead. Another point, mentioned by M. Tanquerel, is very important practically. He says, that those who present the first physical signs of the action of the poison of lead, as the blue line in the gums, and the yellow tinge of skin, appear for a time to be quite well; all the functions are correctly performed; the subject complains of no pain, and follows his employment as usual.

The nervous lesions which ultimately occur, assume all the forms of delirium, coma, epilepsy, &c., sometimes after several attacks of colic, sometimes unpreceded by it. After the occurrence of these, especially the epileptic seizure, M. Morel says the reason is never again sound. We do not enter further into the subject, as it belongs more especially to toxicology proper.

The influence exerted by diseases of the *cerealia*, ergot of rye, maize, &c., upon the degenerations of the race is very important. In introducing this subject, M. Morel, illustrating his position chiefly from the epidemics of ergotism in 1769 to 1772, takes occasion to point out that epidemics are not isolated facts, but are intimately connected with various widely extended cosmical changes. He attributes the disease of the grain to wet seasons, and alludes to the floods and inundations, the earthquakes and electrical phenomena, the fogs, and the immense amount of insect life in those years. The disease of the rye causes ergotism in its various forms; and, as this is not a constant occurrence, but only a casualty, the disease is not endemic, like the pellagra, which is the result of a constant degeneration of the maize. The former may be considered an acute epidemic; the latter is essentially chronic in its nature, and endemic.

The connexion of the convulsive affection called ergotism with the diseased rye, is thus indicated by Taube:—

1. All the persons attacked had eaten rye meal. 2. They experienced immediate amendment on change of diet. 3. They constantly relapsed on returning to that kind of food. 4. The rye of these years contained a very large quantity of *ergot*. 5. This ergot appeared to be more powerful in its effects than in other years. 6. The rye itself was altered, and appeared to possess some of the properties of the ergot.

Four forms of ergotism have been noticed—the mild, the acute, the chronic, and the gangrenous. The mild, or benign ergotism, attacked almost the whole population of the districts visited in Germany. It was characterized by formications in the feet and hands, with a vague condition of anæsthesia and deafness; and gastric irritation, with tendency to diarrhœa and vomiting.

The acute form was similar in many respects to lead colic. There occurred blindness and fainting, trembling of the limbs and cramp, and violent spasm of the flexor muscles. There was great precordial oppression, and intolerable griping; spasm of the glottis and cold sweat. Speech and sense were abolished. About the third day death took place, and no instance of recovery from this form was known. Two remarkable circumstances may be noticed in connexion with this violent convulsive affection. The pulse remained unaffected; and the milk in the breasts of women appeared to have no ill effects on the child.

In the chronic form, for some days before the complete formation of the attack, the patient experienced a feeling of weight in the limbs, precordial tension, dislike for food, and cold in the trunk and vertebral column. There were occasional twitchings and cramps, constant retraction of the tendo Achillis, and the formications extended to the internal organs. At this period there were no functional disorders; the action of the bowels and skin was normal. Then occurred a few hours of suffering, similar in nature to those of the acute form; after which there was extreme prostration, followed by some peaceful sleep, and an awakening with some sensation of desire for food. The intervals of the attacks presented always the same characters, viz., insensibility of the extremities, formications, trembling of the limbs, derangement of vision, tension in the precordial region. This last symptom announced the recurrence of the acute stage. Sometimes spasm and convulsion alternated with a cataleptic condition, which was generally the transition to epilepsy, succeeded by delirium. A sardonic laugh preceded the intellectual disorders; the memory was lost, and the most fierce mania succeeded. This was generally fatal in the acute stage; when it was not so, the patients fell into marasmus and intellectual torpor, from which many never recovered. The few who did recover had an almost interminable convalescence.

Attributable to the same cause, the disease of the rye, is the terrible affection known in France for many centuries as the *mal des ardents*, the *peste noir*, the *feu de St. Antoine*, or more recently, recognising its source, gangrenous ergotism.

"The unfortunate victims of this malady suffered most intolerably. The grinding of the teeth, the contortions of the whole body, the terrible cries, indicated the most inexpressible agony. They complained of a fire under the skin, which consumed the muscles, and separated them from the bones; yet the surface was cold, and it was difficult to communicate any warmth. Later, the parts affected appeared like charcoal, and the air was poisoned by the smell of the putrid flesh separating from the bones. The arms and legs came off completely from the trunk; the same affection seized the internal organs, and they perished in extreme agony. In some cases the malady stopped short of gangrene; but this was a rare exception, and fever succeeded. In some cases there were cramps and convulsions."

The fatality of this fearful affection varies. In the mildest epidemics, half the attacked died; in others, the mortality was general. In the epidemic of 1099, none escaped who were once affected; in 994, 40,000 individuals died of it in the South of France. M. Morel adds—"Il est inutile d'ajouter que l'on ne connaissait aucun moyen medical contre cette maladie." Dr. Salerne, of Orleans, relates the case of a child ten years of age, whose thighs detached themselves from the articulations without any hemorrhage; his brother, aged fourteen, lost the leg and thigh of one side and the leg of the other. Both died after twenty-eight days! Amputation was of no avail—it seemed rather, in many cases, to hasten the fatal result.

The consideration of these diseases sheds a gleam of light, lurid though it be, upon many of the fearful epidemics of the middle ages, the causes of which have been, and still are, hidden in so much mystery. In tracing the source of the maladies under present consideration to the change in the principal article of food amongst so many millions, and reflecting also that rye is by no means the only grain susceptible of such a morbid transformation as to become poisonous; perceiving also the points of analogy between these affections and many others from time to time devastating large districts, we cannot fail to be struck with the important bearing which this subject has upon general hygiene, and the urgent and paramount claims for its consideration, medically and administratively. This will receive still further illustration from what remains to be said on the subject of *pellagra*, an endemic and chronic disorder, arising from a degeneration in the maize, the staple article of diet of large districts. The following case is intended as an illustration of the results of a diet almost solely consisting of this grain, which, it must be understood, is in these northern latitudes always more or less *imperfectly* developed; hence the chronic and endemic nature of the resulting disease.

An agricultural labourer, F——, aged thirty-five, married, and the father of many children, pursued his occupation, with his family, in the environs of Brescia. Their condition was that common to the Milanese and Venetian peasantry. Their nourishment consisted almost exclusively of vegetable food—chiefly maize, or cakes of rye or millet. Meat and salt fish were very rare events, and only eaten as preparation for the most arduous duties. The country was salubrious; the water pure and abundant; there was no endemic affection but pellagra; but this attacked nearly one-sixth of the entire population. The family of F—— had not been spared; his father had died in the last stage of pellagrous marasmus, and his mother was affected with it. Many of his brothers and sisters had suffered from the same affection; he himself had been in the army, and thus removed from the cause; but scarcely three years after his return home he began to experience the precursory symptoms.

Every spring brought much gastric disorder; disgust for food alternated with gnawing hunger, and constipation with diarrhœa; there was a constant bitter taste in the mouth, nausea, and occasional vomiting. Such were the preliminary troubles. There succeeded, about the spring equinoxes, the cutaneous affection peculiar to this disorder—a kind of desquamative erythema of a very painful character on the back of the feet and hands, the fore-arm, the forehead, and the cheeks.

The nervous system, meanwhile, was much depressed and disturbed; there was extreme lassitude, and singing in the ears, with stupor; and after this violent pains in the spine, and especially in the sacrum. By the summer solstice these disturbances chiefly passed away, and the amendment each winter was great. So passed the first period of the malady.

In the second period, the affection of the skin changed character, and resembled some forms of ichthyosis, preceded by vesicles and bullæ. In some cases horny vegetations appeared on the forehead. The affections of the nervous system were now very grave; they were, frequent stupor, pains in the head and spine, involuntary spasm of the muscles of the back of the neck, obscurity of sight, double vision, amblyopia; trembling, cramps and spasms of the extremities; delirium, hallucinations, shaking of the head, and jactitation. Various forms of excitement and depression alternated for some time previous to his sinking into the condition of mind specifically belonging to pellagra—that of *melancholy, with suicidal tendency.* Had he not been perpetually watched, he would have thrown himself into the water. Meanwhile, uncontrollable diarrhœa induced extreme feebleness —and so terminated what is called the second period.

The third and last period was marked by an aggravation of the previous symptoms. The fever was continual, the stupor profound, the emaciation extreme and progressive. Diarrhœa and general dropsy continued; and finally some convulsions occurred, followed by death. Thus terminated seven years of suffering, with rare intervals of relief.

The principal pathological changes found after death were softness and friability of the mucous membrane; intestinal ulcerations; general softness of the substance of the brain and spinal cord; injection and adhesion of the meninges; and some effusion in the ventricles. The skin in many parts was like leather; the epidermis six times its normal thickness; the nerves appeared more voluminous than natural, and serosity exuded from a transverse section.

We have detailed at length this typical case of pellagra, because in addition to the light that it throws upon the nature of the individual affection, it is not without a most serious significance in relation to the subject of imperfect, insufficient, and exclusive nourishment in general. The analogy between the production of disease in this form, and that resulting from diseased potato tuber will readily suggest itself. There remains one further consideration affecting the race, of still more extended importance.

In lead poisoning the individuals affected are too few, comparatively, and the fatal termination too sudden, to permit any very definite calculation as to the effect finally upon the race. In that resulting from the ergot of rye, its occurrence as an acute and occasional epidemic only, has the same negative result.

But in the poison which produces pellagra, the causes are permanent, and "affect compact populations subjected for ages to the same degenerative influence." They act both by direct poisonous influence, and by the ever-increasing hereditary taint.

"The degenerate races have time to produce themselves in accordance with the fixed and invariable laws which preside over the formation of organized beings generally; and pellagra must be classed amongst those maladies which, by being transmitted from generation to generation, perpetuate those special types of cachexia and degradation which can no longer propagate the great human family in the conditions of its normal development."—(Morel, p. 246.)

M. Morel generalizes upon the action of the preceding poisonous agents as follows:—

"The reader, who has followed with attention the developments upon which we have entered, will have been struck with the analogy which the principal poisons present in their ultimate effects upon the nervous system; excepting those whose action is so energetic as to be very speedily fatal. Formications in the extremities, anæsthesia and partial paralysis, and transitory delirium, invariably precede those convulsive conditions which are the *avant couriers* of general paralysis and the complete loss of the intellectual faculties. In a word, the regular progression observable in the organic lesions permits us to define the phases through which individual degeneration must pass before attaining its extreme period. It is clearly demonstrated that there exists a class of beings whose intellectual, moral, and physical degeneration must be attributed to the pernicious influence of poisonous agents."

The influence upon future generations is indicated by a passage quoted from Dr. Buchez:—

"No one is ignorant that many organic dispositions in the human race are transmissible from one generation to another; but it is not generally known how far this principle extends. It is believed in general that form and appearance are transmissible, but it goes much further than this. It is ascertained that *all* morbid dispositions, all *pathological predispositions* are inheritable from parents to children, as well those belonging to the organs of vegetative as of animal life. The predisposition to nervous maladies, to epilepsy, to mania, is transmissible as well as that to gout, rheumatism, scrofula, dartre, &c. *Now these predispositions have not constantly existed in all preceding*

*generations, but have been acquired by some part of the ancestry, and handed down to the descendants, the morbid taint becoming more and more pronounced in every generation.*"

M. Morel adds—

"Whatever may be the form of the physical degradation, and whatever the nature of the lesions experienced by the individual, whether arising from alcohol, opium, or other causes, it is *not necessarily* the same typical form, nor the same lesions, which are to be expected in his descendants. The deviation from the normal type of humanity shows itself in succeeding generations by internal and external signs perhaps much more alarming; since they represent enfeebled faculties, an addiction to the worst tendencies, and the limitation of *intellectual life* to a certain period, beyond which the individual is no longer in condition to fulfil the functions of humanity. In contemplating successive generations under these unhappy conditions, we observe a series of proteiform nervous phenomena, having in general a convulsive type; and forming those etiolated, suffering and morbid temperaments, as well as those incredible moral perversities and intellectual aberrations, which by their nature and frequency justly astonish those who have not watched intently the formation of such degenerate races."—p. 324.

M. Morel proceeds to show elaborately how these phenomena are to be studied in reference to their *cause*, rather than to their pathological lesions, which are, in truth, in a great proportion of cases absent. He dwells long upon the *circular* and self-reproducing character of these degenerative affections; but this part of the argument we omit, the subject as yet not appearing sufficiently ripe.

M. Morel attaches this subject of degenerations to the study of mental alienation, as follows:—

"In proportion as I advanced in the career which I had adopted as a speciality, I was not long in perceiving that the curability of mental affections became a problem more and more difficult of solution. The strange complications supervening upon very simple cases of delirium—the frequency of relapses—the circle of successive transformations fatally traversed by those affected with certain forms of mental disease—lastly, the *almost constant want of relation between the gravity of the symptoms and the anatomical lesions*, and the ever-increasing proportion of incurable cases;—all these became to me facts too often repeated not to have their reason in the intimate nature of the evil to be combated. . . . I received communications from my scientific brethren; they were unanimous in recognising the complexity of the causes, and the extreme difficulty of properly meeting them. Never since the origin of medical institutions had such strenuous efforts been made for the interests of the insane. How was it, then, that, in reference to cures, these efforts were so disappointing? . . . Can we admit that the predominance of idiopathic affections of the brain was the cause of such want of success? Certainly these are ever on the increase, but the difficulty is not removed by the acknowledgment; for *why are they* on the increase? I only saw one mode of accounting for the fact, which was, to consider, in the generality of cases, mental alienation as the final result of a series of moral, physical, and intellectual causes, which, by determining in man successive transformations, connect him with the morbid varieties of the race, which we have called degenerations. In this view, those so affected only represent to the mind those departures from the normal type which are not only incapable of perpetuating the human chain in its integral condition of *capability of progress*, but are the greatest obstacle to this progress by their mere contact with the healthy part of the population."—p. 344.

Mental aberration, serious as it is in any point of view, in this light becomes doubly so, when it is not merely an individual lesion, but the fatal climax, and, as it were, the *resumé* of a long line of individual and hereditary affections. It is easy to conceive how, from one generation to another, the moral and physical condition is gradually deteriorated, when what was the habit merely of one generation became an instinct and impulse in the next; when added to the hereditary taint was the force of example positively, and negatively the absence of all instruction and useful education; when to the disease of mind already existing, either actually or potentially, was systematically denied the exercise of the commonest rules of hygiene or therapeutics, and the ordinary restraints of morals and religion. In cases representing so deplorable an ancestry as this, medicine will do little in altering the condition of the individual, which may be considered virtually unmodifiable; but there remains a noble part to play in the enunciation of principles which, when carried out, will tend to the removal of those causes to which so many of these evils are attributable. It is true, as already seen, that degeneration tends ultimately to the extinction of the degenerate race; but this is not enough. The death of the branches of a tree is not sufficient to regenerate it, when its roots are fixed in a permanently unhealthy soil. Whether the human race as a whole is in a state of degeneration or not, is not the question—perhaps, were it so, it might be insoluble. But it is clearly proved, or proveable, that a great number of degenerations are in progress in the species, and that these are in certain proportion to well-defined causes. These causes are in some degree removeable; in other respects, owing to the constitution of society, they admit only of more or less modification. Be this as it may, the first step in the process is to point out the source of these evils, and the mode in which they first act upon individuals, and, through them, upon society at large.

M. Morel considers that each of these causes of degeneration results in a fixed specific type or seal (*cachet*), ultimately expressive of its cause, however various the intervening manifestations may be; and that, in a more advanced stage of the science, each will be as recognisable, and as distinctly to be diagnosed from any other, as is now the cretin type (i.e., the degeneration from geological causes) from any other, as that from opium or alcohol. The time for this scientific classification has scarcely arrived as yet.

From these general considerations we pass to a more extended application of the subject. Having traced the effects of poisonous agents on individual organism, and on the descendants of those so affected, it remains to apply the same and other modes of investigation to large masses of people—to have recourse to statistics, to history, and comparison of facts, to ascertain the intellectual, moral, and physical condition of a society in which such evil influences may be rife. We quote M. Morel :—

"When, in a society, a people, a race, we find that the moral and intellectual powers have undergone considerable degradation; that maladies up to a certain time unknown, now have a serious influence on the public health; that the number of insane persons and of criminals increases in great proportion;—we have a right to conclude that a cause producing certain results in individuals and families is likely to do the same in societies. I am about to apply this method of investigation to a country concerning the intellectual, moral, and physical condition of which I am better informed than upon others—I mean Sweden."

The abuse of alcoholic liquors in Sweden appears to have begun in the last century. In 1785, Dr. Hagstrom, struck with the growing evil, made an energetic appeal to his fellow-citizens to check a vice which was not only an outrage to religion and morals, but which threatened seriously future generations. Since then "thousands of voices have been raised to the same end," notwithstanding which the evil has increased to such an extent that Dr. Magnus Huss writes :—

"Things are come to such a point, that if some energetic means are not adopted against so fatal a custom, the Swedish nation is menaced with incalculable evil. The danger is not future and contingent; it is a *present* evil, the ravages of which may be studied in the present generation. No measures *can be too strong;* it is better to save at any price, than to have to say, *It is too late.*"

It is unnecessary to enter into the detailed history of the rapid introduction of alcohol into Sweden. An analysis of the statistics bearing upon the point proves the startling fact, that there are one million and a half of persons (half the population) who consume annually 80 to 100 litres (140 to 175 pints) of brandy each person. We need not be astonished at the opinion expressed by Dr. Huss, that Sweden is threatened with irremediable destruction ; nor at the fact stated on the same authority, that the people of Sweden have already *degenerated* in stature and physical strength. But this is not all. New diseases have invaded the country ; chronic gastritis and scrofulous affections have appeared in frightful numbers; and chlorosis, according to Scandinavian physicians, formerly unknown, attacks now all classes, rich and poor—the dwellers in the country as well as in the town. The hereditary tendency to drink, combined with the constant example, produces a powerful influence ; children of twelve, ten, or eight years evince already the evil predilection.

The duration of life in Sweden seems much affected by alcoholism. The city of Erkistuna is one of the places most addicted to drinking, and the mortality is 3 per cent. annually, whilst that of the entire district is but 2 per cent. ; and in some districts where much less alcohol is consumed, as in Jamtland, the mortality is but 1 in 80.

Mental alienation appears to be considerably on the increase ; and suicide occurs so frequently, that we could wish to suspect the returns of inaccuracy. In ten years, the average of suicides of men between twenty and fifty years was 1 in 57 deaths. This is enormous ; "but," says Dr. Huss, "if we reckon as suicides those who have died of the immediate effects of alcohol, in a state of intoxication, the proportion will rise to 1 in 30 deaths."

Crime is frightfully on the increase. In the year 1830, the proportion of criminals convicted of various offences was to the entire population as 1 to 143 ; in 1845, the ratio was 1 to 100.

M. Morel proceeds to signalize the evils resulting from the same practice in his own and other countries. He mentions, in passing, that in the United States from 40,000 to 50,000 persons die annually from the effects of alcoholic liquors. He does not entirely deny that in some cold climates, and under certain conditions of nutrition, alcohol in some proportion may be a necessary article of diet ; but he adds, with M. Quetelet—" Quand un climat crée un besoin, il est bien difficile que l'homme n'en fasse pas un abus."

He thus sums up the conclusion :—

"We have need of no further proof to show that the abuse of intoxicating liquors produces the same disastrous results in nations as in individuals. The effects are the same in all latitudes ; but they are produced more suddenly and forcibly in proportion as there exist other causes of degeneration, and as the less degree of civilization is unable to develop, as a counterpoise, the salutary influence of morals and education. Under a cause of degeneration so strong, new maladies are developed, and the old ones assume a more serious aspect. The average duration of life diminishes, sterility increases, and the viability of children is more uncertain, whilst the intellectual and moral disorders are signalized by the ever-increasing numbers of the insane, of suicides, and of crimes."—p. 392.

The degenerations due to the use of opium and other narcotics are more difficult to trace statistically, although no less indubitable. Our knowledge of facts, past and present, is more limited; but there is another reason still stronger existing in the fact, that such statistics as we do possess, concerning moral offences, cannot be viewed as equally significant with the records of crime in western nations; seeing that many of those acts, which, amongst us, are referrible to crime or mental alienation, are, amongst the Orientals, to be considered as attached to mistaken notions of religion or morals, or as originating from peculiar legislative enactments. To take as an instance, suicide; it is certain that this crime is extremely frequent in China; yet it must not be considered as indicative of the same amount of mental alienation in society which an equal average amongst ourselves would show:—

"It is almost impossible to imagine," says the Abbé Huc, "the readiness with which the Chinese commit suicide. The merest trifle, or a word, induces them to hang or drown themselves, the favourite modes of suicide. In other countries, if a man wishes to revenge himself on his enemy, he kills him; in China, he kills himself. The reasons for this are manifold; first, the Chinese legislation holds *him* responsible for the suicide who has been the cause or occasion of it. In killing himself, therefore, a man throws his enemy into the hands of the executive, who torture him, ruin him and his family, and perhaps take his life; and the family of the suicide ordinarily obtains large damages. On the contrary, by killing his enemy, he exposes himself, his friends, and his family to ruin, and deprives himself of the rites of burial. Again, the suicide, instead of being viewed with horror, is honoured as a brave man; and, lastly, it appears that the Chinese fear many parts of their judicial processes more than death."

In like manner, the great frequency of infanticide attaches itself rather to mistaken views of demon worship (according to the same authority) than to any actual criminal propensity. It is thus evident how differently the statistics of crime must be interpreted in reference to the nations of the East. M. Morel also considers that vast as is the evil of opium smoking in China, it has been in some measure exaggerated; otherwise nothing but the extinction of the race could result. The prevalence of tobacco smoking is also an argument against the universality of the other practice; as it seems ascertained that opium smokers find no pleasure whatever in tobacco. M. Huc says that the use of tobacco has become universal in the empire. Men, women, and children all smoke, and almost without cessation.

Whatever the employment, smoke accompanies it. If they pause in eating, it is to smoke; if they awake at night, it is to light a pipe. Whatever causes combine to produce the result,

"There exists undoubtedly a tendency to decay in the moral, physical, and intellectual condition of this people; and, as the extraordinary abuse of poisonous agents, such as opium, exercises so powerful a deteriorating influence upon individuals and families; on the principles already set forth, it is rational to conclude that the effect on the race will be analogous. Were it otherwise, the degenerative effect of alcoholism, hereditarily considered, might be open to doubt; but this has been by facts much too serious and weighty; and we cannot but recognise the striking analogies between the consequences of alcoholic poisoning, and that from other narcotics." (Morel, p. 400).

M. Morel considers that great as is the evil attendant upon opium smoking in China and the East generally, it would be very much greater if the practice were introduced amongst the western nations.

"It is reasonable to suppose that the predominance of the lymphatic temperament, the inferior development of general sensibility, the greater indolence and apathy of the Orientals, and the absence of those motives for over-excitement of the functions of the brain found in Europeans, produce a notable difference in the action of any given poison on the human economy."—p. 407.

These considerations naturally lead to the study of *mixed causes* of degeneration; or the influence of those already alluded to, when conjoined with, or modified by, climatic influences, and all the elements which constitute the general hygiene of nations,—their relations as conquerors or conquered,—their habits, their occupations, their manners,—and we may add, *their diseases.* M. Morel's introductory observations on this branch of the subject are interesting and important:—

"It is from not having comprehended these ideas, so simple in appearance, that in their relations towards the people of the New World, Europeans have generally failed in their mission of civilization. Thus it has happened, that instead of assimilating the aborigines to themselves by the intellectual and moral element, which tends to regenerate races, and to raise them from their decayed condition, they have imposed customs upon them, incompatible with the *infantile* condition in which they were found; they have developed in them desires dangerous to satisfy, and appetites of the grossest character. . . . It is sad to confess that the anthropological science of the 18th century has contributed to this result by determinately classing these races as a *distinct species*—races whose differences ought to be examined only with reference to those causes which have modified naturally (or morbidly) the one primitive type. . . . The contact of the people of the Old and New Worlds has been attended generally with such unfortunate results, that many authors consider that when two forms of civilization are in presence, assimilation cannot take place under the ordinary conditions of progress in humanity; and thus they explain the extinction of many American races, and the return of others, once civilized, to a savage life, with instincts more depraved than before. They find further, the proof of this in the presence, in the midst of Europeans, of the melancholy remains of ancient races never completely assimilated to our forms of civilization; or who have only adopted our vices, and become affected with our diseases."—p. 410.

"But if the contact of Europeans has been pernicious to these races, when the sole elements of civilization have been the interests of commerce, and the introduction of vicious habits, it is certain that these in their turn have felt the evil influence of the contact of the Orientals, only borrowing from them their effeminacy and luxury. The influence of climate alone does not sufficiently explain the modifications which the European races have undergone when transplanted to the Indies, to Africa, or to Asia. It is necessary to examine these changes in the new conditions brought about by conquest, by colonization, by immorality, in short by all which I have included under the head of *mixed causes of degeneration.*"

The history of the Spanish and Portuguese conquests abound with illustrations of this opinion. The conquered races have well nigh disappeared, whilst the conquerors have greatly degenerated; and their mixture with the aborigines has produced a degraded race, which presents no element of perfectibility in the future. For instance, in Malacca there remain 3000 descendants of the old Portuguese conquerors. Their fathers were the companions of Vasco de Gama and Albuquerque; yet they are in a state of utter degradation, even as compared with the aborigines, amongst whom they dwell. They bear chiefly great names, but they have no idea of their ancestry, or their glorious deeds—even tradition is lost. Their degradation presents itself under its characteristic forms—stunted growth, physical ugliness, default of viability in the infants, obtuse intelligence, perverted instincts, and a succession of progressive morbid transformations, reaching finally the extreme limits of imbecility. Dr. Yvan, from whom these details are chiefly drawn, adds that they are in the most frightful destitution, living almost promiscuously, like wild beasts; they do not till the ground; they live without any social laws; they have no priest, nor any form of legislation. They have no idea of time, and appear incapable of conversation. The men smoke, and the women chew betel-nut, "tenant suspendus à leurs mamelles affaissées quelques avortons débiles." M. Morel adds:—

"It is impossible to find a more striking example of degradation of the species. It shows us the terminal phase of some hereditary mental affections in families. The fatal chain of pathological phenomena finishes by inducing in the last descendants a state of imbecility and idiocy incompatible with the normal propagation of the human family."

In inquiring into the causes of this degeneration, we first meet with the crossing of the races, to which the Portuguese have shown less antipathy than either the Dutch, the English, or the French. Climatic agency exerts its powerful influence of enervation; but more than all this, is the fact of the adoption of a system (or rather an absence) of hygiene and morals, simultaneously with the mixture of breed, which belonged neither to themselves nor to the aborigines at first; but has grown out of the despair or apathy of the one, and the luxurious sensuality of the other. As a contrast to the unhappy condition of the race just mentioned, Dr. Yvan draws a charming picture of an isolated company of French colonists, the descendants of the earliest settlers, in the Isle of Bourbon. They have never mixed with any other race; they have preserved a primitive simplicity of manners, and purity of morals; and the result is a physical and intellectual development of a very high order. They appear to be amongst the most beautiful of all people.

M. Morel proposes two further questions for examination—the influence of climate, and that of mixture of races upon the formation of varieties.

Acclimation has been shown to be more easily accomplished, when the efforts of nature are seconded by moral hygiene. But even this is not always successful—the soul is not *all-powerful* in the strife of man with climate. Excessive heat and certain geological arrangements of the soil produce very potent physiological changes. Experience has proved that the first European emigrants who establish themselves in certain tropical regions, *or upon coasts fertilized by alluvial deposits, but insalubrious*, almost all perish; and acclimation only begins to be completely successful about the third or fourth generation. "There are, moreover, some climatic conditions so pernicious, that individuals born in temperate zones have never been able to cultivate, personally, the soil (to turn up the alluvium): and have had to depend for this dangerous labour, always either upon the aborigines, or upon others already acclimatized to other alluvial regions." This was illustrated in the first attempts to construct a railway over the Isthmus of Panama. *All* the Europeans employed in the work perished, and it was found necessary to employ negroes. With regard to these latter, Buffon observes:—

"We only find negroes (indigenous) in those climates where everything combines to produce excessive heat. This heat is so necessary, *not only to the production, but to the conservation* of the race, that in our islands, where the temperature, though high, is not to compare to that of Senegal, the new born negro infants are so susceptible to the impressions of the air, that they have to be kept for nine days in closed and hot rooms; otherwise lockjaw supervenes, and they die."

We cannot follow M. Morel in his interesting investigations into the nature of the degenerative influences exercised by the contact of Europeans with the negro race, as exemplified in the inferiority of the negro of our colonies to the negro original. His conclusions are, that the mixture of the races, not being attended by the exercise of moral hygiene, has produced a degenerate (Creole) race; that they have inherited from their parents nothing but their colour and the spirit of servitude; that they are more idle and sensual than those brought from Africa; and finally, that the intellectual and moral decay of those who have come in contact with European civilization, is a singular and painful contrast with the condition of those who have not quitted their native land. In remarking upon the fact, that hitherto the emancipation of the negroes has not been crowned with success as to the well-being of the race, M. Morel says:—

"Pour nous, qui faisons une étude particuliére des degenerescences dans l'éspéce humaine, nous nous rendons parfaitement compte des insuccés que l'on a signalés. Nos études anterieures nous autorisent déjà à conclure qu'une race dechue et dégradée ne remonte pas *subitement* vers un type supérieur ———."

He, nevertheless, has faith in an extended system of moral training for successive generations, as a means for raising this fallen race to the normal and healthy type of humanity. M. Morel enters elaborately into the examination of the degenerate condition of the indigenous races of North and South America, the Aztecs, the Cherokees, the Hurons, and the other tribes vanished or disappearing before European civilization; also, into that of the Esquimaux, and many others who have been classed by some authors as utterly unsusceptible of civilization, and unamenable to the influence of Christianity. M. Morel will not consider, however, their degeneration (in the sense in which the term is used in this work) as a "fait accompli," so as to necessitate the renunciation of all attempts to re-elevate them to the normal type of humanity. On the contrary, he considers that there is evidence of success sufficient, in our efforts amongst the Caffres and Bosjesmans, to warrant the contrary opinion.

"The work of regeneration, however, is complex, and it is necessary to establish the theory of the formation of degenerate beings, and to define clearly the distinctive characters of *natural* and *morbid* modifications, in order to be able to apply suitable remedies. . . . . . There exists no society where there are not *classes morbidly modified*, whose contact with the sound parts of the population is a perpetual source of danger. . . . In the midst of our own cities there are *veritable varieties*, who possess neither the understanding of the duty, nor the sentiment of the morality of actions, *and whose minds are not susceptible of being enlightened or even consoled by any religious ideas*. Some of these varieties have been justly designated by the title of THE DANGEROUS CLASSES. . . . . . Our ignorance of the distinctive characters of these *morbid varieties*, introduces a deplorable confusion in the treatment. Where moral therapeutics ought to be exercised, there is only the repressive force of the law; and on the other hand, we are found directing the whole activity of our medical powers towards the cure of *unmodifiable beings*, as imbeciles, idiots, and confirmed cretins, who, in general, are not the representatives of any simple and isolated pathological condition, but of the entire degenerative elements of their ancestry."*

As a summary of the deductions from his anthropological inquiries, M. Morel says that—

"We learn that the grand element of renovation for these races is the diffusion of the moral law; and that if their aptitudes have not always been the same for accepting these regenerative influences, and ascending to a superior type, the fault lay often with those who did not take sufficient account of the profound modifications already taken place in the organism; modifications, transmissible hereditarily, and forming the distinctive characters of races and of morbid varieties in the race."

We are duly warned against disappointment resulting from the frequent failures in our attempts to "moralize the masses," on the important principle that as *degeneration* is not a sudden work, but often only reaches its extreme development after many generations, so the work of *regeneration* of truly hereditarily decayed races is not sudden; but that long-continued and well-directed efforts will ultimately be rewarded with success. M. Morel looks most hopefully also upon the advantages to be gained regeneratively by a crossing or mixture of races, notwithstanding the evils already signalised as having resulted from such mixture. He enters in some details from comparative physiology, in illustration of the improvement of breed both in plants and animals attained by this process; and considers that man is not entirely removed from the same physiological laws. But he says with Dr. Buchez, that "it is not sufficient that man be engendered carnally in order to be perfect; he must be engendered spiritually also." As in plants and animals the simple crossing of breed will not succeed without special hygiene in each case; so it must be in man. A specially adapted moral and physical education *must always* form an essential element in any attempts at regeneration by whatsoever process. The evil effects of *consanguineous marriages* are forcibly suggested.

* M. Morel must not be considered as answerable for any apparent lack of sequence in the above observations and quotations. Finding it impossible in our limits to enter with him into his anthropological inquiries, we have selected those ideas which appeared most clearly to illustrate the bearing of his views upon society at large, and those which have a clear practical application, irrespective, in some degree, of the logical course of his argument.

"In the first, perhaps in the second generation, no evil effect may result; but experience shows peremptorily that if continued further than this, even in the rare event of there being no development of hereditary *taint*, there occurs a degradation of the race, a duplication of all infirmities, all the vices, and all the evil dispositions of the mind; heaviness of the faculties, and, sooner or later impotence, with the extinction of the line. . . . These results show themselves not only in families, but in more considerable aggregations of individuals. According to M. Niebuhr, aristocracies reduced to marry in their own circles, are extinguished in like manner. The degenerative tendencies are indicated in these cases under the forms of mania, dementia, and imbecility; and those who have observed the catenation of pathological phenomena are not astonished at the frequency of mental alienation and its hereditary transmission in the noble families of France and England."—p. 526.

Leaving these hereditary considerations, M. Morel now passes on to examine the effects of nourishment either insufficient or exclusive, upon entire races, as he did before in reference to individuals and small communities; and in so doing he contests strongly against the popular opinion that the savage is more hardy and healthy than the civilised man. He quotes Buffon's opinion to the effect that "it is in the action exercised upon the economy by nourishment that we must seek the principal cause of the varieties of form and feature in the human race." He shows that a people living under a civilised government, and leading a regular life, has great advantages over nomadic tribes, or those where every individual must provide for his own support, and alternately suffer hunger, and the effects of an excess of food often bad. If these latter had any advantage over the former it might be looked for in the force or endurance of their bodies. There might also in such tribes be found a much smaller number of defective or deformed persons, and this for an obvious reason. In civilised society the blind and otherwise helpless are cared for by the community; and moreover the character of the mind is of more importance than the formation of the body. But amongst savage tribes, where each individual lives by his own corporeal qualifications, those who are feeble and imperfect perish by the common laws of nature. (Buffon.)

The same author continues thus:—"gross, unhealthy or ill-prepared nutriment causes the human race to degenerate; all the nations that live miserably are ugly and badly formed." "Applying then," says M. Morel, "these principles to our own societies, we find that the air, the earth, and above all, the food, exercise great influence upon the form of men, plants, and animals." Those people who live almost exclusively upon vegetable food, and that in insufficient quantity, are less vigorous, and can support less fatigue than others; and the proportions of the limbs are altered. The Hindoos have the arms longer and much less muscular than Europeans; and it has been remarked that the handles of their sabres are too small for English hands. (Prichard.)

M. Morel examines the force of an objection made to these views, from the fact of the very scanty nourishment of certain religious orders (as the Chartreux) not being attended by any increase of disease; and also from the nature of the affections which do occur amongst them requiring depletory treatment. It is certain that under no circumstances do they eat any meat; and only during six months in the year are they allowed small proportions of milk, fish, cheese, and eggs. Their principal diet consists of pulse, roots, potherbs, &c., dressed with butter or oil. They have but two meals a day, and only one from September to Easter. M. Morel shows that the regularity of life and manners, and the necessary freedom from many vices, tend to counteract the depressing effects of such a regimen. But more than all this is the careful sifting of the candidates for the order. There is a year of probation; and if, in any particular, the novice is found unable to bear the severity of the discipline and regimen, he is rejected. Thus only those are admitted who are of strong, vigorous constitution, and have demonstrated their power to endure privation.

Closely connected with this subject of *insufficient*, is that of exclusive, nourishment, and particularly that by the potato. M. Morel, though acknowledging the great benefits derived from this tuber, considers that its exclusive employment in many countries has exercised a most baneful influence upon the population. Dr. Huss attributes a considerable proportion of the endemic Scandinavian maladies to this exclusive diet; and congratulates himself that the potato disease has compelled the inhabitants to return to the cultivation of other alimentary plants, which they had too much neglected. M. Morel asks:—

"Can it be true that the augmentation of scrofulous and rachitic affections may be attributed to the over-use of this vegetable? Haller, Kortum, Weber, Neuman, and many others, answer in the affirmative. It is related as a curious fact, that the aborigines of New Zealand had no knowledge of scrofulous affections up to the time of the introduction of the potato; but, after that, they have been cruelly tormented thereby. . . . M. Zokalski states that the cultivation of the potato in the interior of Poland has contributed to repulse the *plica polonica;* but that, on the other hand, scrofula has much augmented."

This is by no means conclusive, notwithstanding. The New Zealanders, concurrently with this new culture, received from the Europeans smallpox, syphilis, and the abuse of alcoholic liquors—facts which must weigh heavily in any calculations of causation. It is probable also that rachitis was a common affection in England long before the general cultivation of the potato. It is possible that, in all countries where scrofula prevails, a very exclusive use of the potato may have tended to aggravate its ravages, and to produce general deterioration; but it is going too far to attribute the whole evil to such a source.* M. Morel is also of this opinion, and quotes M. Huss to the same effect.

A singular fact is related concerning a province in Sweden where the potato was almost exclusively cultivated, and where the usual signs of degeneration were remarked. The potato disease having deprived them of this aliment, they were thrown upon bread and leguminous vegetables in very insufficient quantities, and much misery was the result; yet, from the time that the use of the potato ceased, there was a visible improvement in the aspect of *the young children.* Their pale and puffy faces and tumid abdomen became less marked. There was an appearance of some colour in the cheeks, and their former habitual air of stupor gave place to the animation and gaiety which are characteristic of infancy. (Vachtmeister.)

On the propagation of degenerations hereditarily, the author thus explains himself:—

"We do not understand by 'hérédité' simply the malady of the parents transmitted to the children, but the transmission of *organic dispositions.* The precise malady may not be reproduced in the children; but these are endowed with an unhappy organic predisposition, which becomes the point of departure for pathological transformations, whose catenation and mutual dependance produce *new morbid entities* of a moral or physical order, and sometimes of both. . . .

"Alienists have more frequently than any others occasion to observe this. They know that a simple *neuropathic* condition of the parents may originate in the children an organic disposition which develops itself in mania or melancholia—affections which, in their turn, give rise to others more serious still; until in the last links of the chain the whole series is reproduced in idiocy or imbecility. I have constantly observed that the children of an insane father or mother presented from their earliest infancy nervous anomalies, which were the most certain signs of ulterior degeneration, unless steps were taken to avert the danger. The peril is more imminent still when both parents are liable to such affections. In such cases, the first moral or intellectual manifestations of the offspring are often the indices of the gravity of the inherited curse. I have found no other name for such conditions than *instinctive mania.* . . . A law which I believe to admit of

* M. Schleisner attributes scrofula to a transformation of the syphilitic taint. Given, says he, a tertiary syphilis, it is certain that in the second or third generation, we shall find scrofula appearing. A popular prejudice in Sweden attributes the spread of scrofula to vaccination. But in Iceland vaccination has become very general, and scrofula is almost unknown in that island, except in one small district.

no exception, is that which indicates the profoundly perilous state of the children of those who, on the one hand, have inherited physiologically evil organic tendencies; and, on the other, were born under the sad influence of the immoral and vicious conduct of their parents. This is the law of *double fecundation*, physical and moral."*

M. Morel proceeds to review generally the indications of cure, and the manner in which we must comprehend the regeneration of the species, amongst the victims of the poisonous agencies above discussed. He views these in two aspects; the first, those who are in the acute stage of the affection, and are generally placed in asylums or other establishments specially adapted for them; the second comprises that class, perhaps more numerous, who, having run through the circle of the morbid transformations already described, either at their own homes or in the hospitals, end their career in marasmus and chronic cachexia; not without having handed down to their descendants the germ of the degeneration by which they themselves are affected.

Much has been done both by science and by the legislature for the relief of both forms of affection: yet it must be confessed that therapeutics have not advanced so rapidly as might have been expected from the numerous and strenuous efforts made. M. Morel attributes this to the backward state of the science of causation. From looking upon these affections too much in the light of isolated pathological phenomena, has arisen the over use and want of success of purgatives and the antiphlogistic method generally.

Since a better and more extended comprehension of the cause and *representative* nature of these affections has arisen, the delirium, the agitation, and the fury, which used to be treated by repeated bleedings, are better understood; and are met by tonics, and such regenerative physical and moral means as can be applied to those who are not in the last period of the degenerate condition, that of dementia or general paralysis.

Pellagra itself, a degeneration from diseased food, used to be treated by copious bleeding; but a recognition of the cause led to the adoption of methods of a more strictly hygienic and pro-

* Alcoholic intoxication and its results afford a noteworthy example of this double fecundation in respect both of the moral and physical nature. The reason is plain, when we consider that the fact of drunkenness in the parents is indissolubly allied to numerous degenerative conditions, which create misery, immorality, and, so far as regards the children, the absence of all proper moral or physical hygiene or religious culture. "I have constantly found," says M. Morel, "the descendants of the sad victims of alcoholic intoxication in their chosen habitats, the asylums for the insane and the houses of detention. I have constantly observed in them deviations from the normal type of humanity, revealing themselves, not only by arrests of development and anomalies of constitution, but by those vicious dispositions which seem to inhere in these unfortunate creatures. I am convinced, from these observations, that the victims of this double fecundation rarely, if ever, escape the above-stated consequences."

phylactic nature, involved in separation from the diseased grain. A sad experience, however, showed that those who were cured, relapsed very shortly after returning to their pristine diet; and the relapse was always worse than the original disease. A new era arose, therefore, in treatment,—"to prevent those maladies which it is often impossible to cure, seeing that their causes, operating sometimes permanently and sometimes periodically, constitute in either case a source of immense danger for the future of the human race."

"It was a spectacle worthy of admiration—that of the zeal, devotion, and self-negation, which physicians evinced in the search for and application of preventive means. Whilst the immense impulse given to all branches of industry and commerce struck like a vertigo upon the inhabitants; whilst the thirst for gain, on the one hand, and the imperious necessity to live, on the other, precipitated every one—masters and workmen, into that exaggerated course, where so many left their health and reason; the physicians watched over the dangers of the situation, pointed them out in their works, and sought for all the means of combatting the dangers which they foresaw. . . . . It would be impossible to enumerate all that has been done in this way. The natural sciences, chemistry, physics, physiology, all lent equally their aid to the elucidation of the question of the deleterious nature of certain occupations and habits. . . . . These researches, we may add for the honour of medicine, have given such an impulse to the science of hygiene, that its influence is ever felt more and more in many social arrangements, and has been sufficiently powerful to modify certain legislative enactments in European countries; and to induce new ones eminently favourable for combatting the action of degenerative causes."

The most powerfully active sources of degeneration are, as we have seen, chronic alcoholism, the insufficiency of nourishment of a proper kind, and the alteration of those cereals most necessary to man. When the growing evils of alcoholism were first noticed in Sweden, naturally the first suggestions involved the limitation of its production by government, and the formation of temperance societies. But M. Huss suggested that great as was the evil, it was not the sole one. The insufficiency and diseased condition of the cereals, the almost exclusive nourishment by potatoes, and the almost entire absence of animal food, ought to weigh heavily in any measures adopted for preventing the degeneration of the species. He proposed a regenerative method, involving the return to a proper cultivation of the grains displaced by potatoes; to introduce into the general dietary a larger proportion of meat; and to encourage the cultivation of the hop, a plant eminently tonic and reparative. These methods, though opposed actively by the ignorance and poverty of the people, and passively by the supineness of the government, promise ultimately to be crowned with success.

In reference to pellagra, an eminently degenerative endemic and hereditary affection, it has been proposed* to call upon government to prevent the marriages of those affected; but it appears scarcely probable that any measure of this nature could be endured in free countries. In default of this, the resources of chemistry have been energetically called upon to devise means for making of the maize (the source of this disease) a bread which will be at once nutritious and innocuous; and this appears from a paper in the *Agricultural Journal* for 1853 to have been at last accomplished. Nevertheless, when science has done her work, the prejudices of the people have to be conquered.

"As to the potato, it is now demonstrated," says M. Morel, "that the disease is nothing more than the result of a degeneration due to carelessness and neglect of scientific principles on the part of the cultivator. *The potato has not been renewed from seed.* Its perpetual cultivation in the same soil has caused ultimately a deficiency in the materials necessary for its nourishment, and the exaggerated employment of soot as manure has produced monstrous varieties, which have quickly degenerated." The remedies are obvious. M. Morel adds:—

"The necessity for better nutrition is still more imperiously indicated, inasmuch as the predominance of nervous affections has created in all classes of society, without exception, physiological and pathological conditions, which deserve in the highest degree the attention of hygienists, and the solicitude of governments. A practice of eight years in a large asylum, containing 1000 inmates, and responding to the exigencies of more than 2,000,000 of individuals, has shown me that affections which I consider eminently degenerative, are spreading ever more and more extensively in the inhabitants of the country. The peasant constitution is more feeble, and habits of intoxication are spreading into districts hitherto preserved from them. Great numbers of young people inaugurate their entry into adult life by the excesses of their parents. Syphilis and scrofula are on the increase; and neuroses, which formerly appeared to be only the birthright of the higher classes, as hysteria, hypochondria and chlorosis, attack now the inhabitants of the country. There results a manifest increase in the number of suicides and of cases of mental alienation, without counting a general impoverishment of the organisms, showing itself by debility, cachexia, and all the attributes of a nervous and degenerate temperament. I am not a pessimist, and far from exaggerating the situation, I only superficially point out the danger."—p. 605.

"Some considerations upon the maladies produced by marshy effluvia, and by the geological constitution of the soil, will complete what I have to say upon the theory which represents poisonous

* Strambio.

agencies as amongst the most active causes of degeneration in the human race."

M. Morel proposes to demonstrate; (1), the strong analogies between the conditions of those living in marshy and those in cretinous districts; and (2), that the extreme state of cachexia known as cretinism has no other origin than that which determines the cachexia of marshes. He considers that it is not by treating individual cases, but by attacking the disease (cretinism) in its causes and origin, that the evil can be extinguished.

As an example of the degeneration of organisms in marshy districts, he quotes the graphic account given by Montfalcon of the inhabitants of "la Bresse," the same being applicable with slight modifications to the dwellers in all marshy districts.

The Bressans, disinherited by nature, only feel the burden of life; the mournful influence of their climate is impressed upon their features; it modifies to an extraordinary extent their functions and faculties. They are born sickly, and they cease to live at what should be the age of vigour. All the elements conspire to the ruin of the Bressan. The air he breathes, the water he drinks are both poisoned; his miserable dwelling is scarce a defence from a pernicious atmosphere; his food is coarse and insufficient; and the kind of labour which he pursues (amid humid forests and morasses) does not permit him to anticipate a brighter future. His stature is short, his bones ricketty, his skin sallow, thin, and unhealthy, his muscles flabby and undeveloped, his features tumid, his belly swelled and dropsical. Scarcely has he quitted the breast when he begins to languish and emaciate; a large proportion die before the age of seven; those who survive do not *live*, they *vegetate*. They are ever subject to dropsies, putrid and malignant fever, passive hæmorrhages, chronic ulcers, and a host of other diseases, which would render life intolerable, were it not for the corresponding apathy of mind. "Melancholy, apathy, a sort of idiocy, is the habitual expression of a countenance rarely modified by passions." Old age commences at forty-five—they are decrepid at fifty-five—few reach sixty. "*We do not live*," said one of these wretched creatures on one occasion,—"*we do not live, we die.*"

Thus the *marsh degeneration* develops itself physically in stunting of the individual, engorgement of the viscera (especially the spleen), languor of all the functions, aggravation of ordinary maladies, complex lesions only explicable from the atony of the nervous system, and finally the short duration of life. Intellectually it manifests itself by torpor of the understanding, apathy, a sort of stupor almost idiotic, and in all cases an indifference of the most profound character.

The children born of such parents are necessarily degenerate; and being exposed to a continuance of the same original cause, are ever progressively deteriorating. The population diminishes, and must finally become extinct, unless supported by immigration. Animals and plants undergo a like decay.

The *marsh*, producing the poisonous miasm, requires for its formation the following conditions:—an argillaceous soil, preventing the filtration of the water; a basin where the waters accumulate, and where organic matter may decompose; and a temperature high enough to determine the evaporation of water charged with a miasmatic principle, more or less deleterious, according to the nature of the putrid matter contributing to its composition.

M. Morel transfers the study of the effects of this poisonous miasm from its natural habitats to the midst of large cities, and finds "the same pathological phenomena evinced not only in the acute forms of typhus and typhoid affections, but in the *etiolation* of the race, and a degradation which yields in nothing to that noticed in marshy districts, and those infected with cretinism."

In illustrating this position, London, and many of our large towns, occupy a prominent and most unenviable notoriety. He quotes M. Leon Faucher in his terrible account of Whitechapel, Mr. Toynbee upon Westminster, Dr. Southwood Smith, and many other of our own writers. The following is one passage out of many similar:—

"The chamber of a person attacked with fever in an apartment in London, where the fresh air does not circulate, is in a condition perfectly similar to an Ethiopian marsh, where heaps of locusts are rotting. The poison is the same, and only differs in intensity. Nature, with her broiling sun, languishing air, and putrid morasses, manufactures pestilence on a large scale;—poverty, clad in rags, and steeped in filth, excluding the air and increasing the heat, succeeds but too well in imitating nature."

Liverpool, Manchester, Wolverhampton, and many other towns are passed in review, with statistical details of a frightful nature—all those revelations with which official inquiries of late years have made us so familiar. It is time, indeed, that these revelations should be followed by energetic measures for radical reform, when the very hearts and foci of our wealth and enlightenment are known to our continental brethren chiefly as the hotbeds of fever, pestilence, and general degradation.

But the malaria of large cities producing these fearful results does not *act alone*. The absence, insufficiency, or impurity of nourishment—the abuse of alcoholic liquors, and sensual pleasures—the absence of all intellectual and moral culture, play a fearful part in the degenerative progress. After dwelling upon the insalubrious nature of some employments, and their relations particularly to the young, chiefly of our country, M. Morel proceeds to sketch the intellectual and moral condition of the working masses, having defined the deterioration in physical constitution as analogous to that already described as existing in marshy districts. Referring chiefly to Wolverhampton, he says:—

"The education of children is literally nothing; the child of five nurses the child of two years, and the child of seven watches over both, and keeps the house in the absence of its parents. To facilitate this, the mothers administer to the nurslings preparations of opium. . . . Another pathological phenomenon manifests itself which we believe to be inevitable in the morbid degenerations of the species—that is, the *arrest* of *development* of the intellectual faculties. Their intellectual existence is limited to a certain age, beyond which not only the evolution of the faculties remains stationary, but the children who have been able to learn forget irremediably the few ideas they have acquired. . . . We have had occasion to observe the same in all localities where cretinism is endemic. Children who *appeared* intelligent experienced this intellectual *arrest*, which almost always corresponded to one of a physical nature. These children become the victims of undeserved punishment: they *cannot learn*—they are not culpable—they are only undergoing the inevitable consequences of congenital degeneration.

"The moral condition of the people is no less sad. But one remarkable fact is developed by the inquiry,* that, notwithstanding the general corruption of manners consequent upon drunkenness and unnatural accumulations of people in confined lodgings, there are but few instances of seduction, and few natural children. *La pauvreté du sang, la maigre chère, et l'epuisement qui suit le travail, ne laissent aux jeunes filles ni temps ni force, ni desir pour le mal.* And thus the unfortunate creatures are protected against the consequences of vice by the very excess of their sufferings! But the corruption of the soul is there, though the prostitution of the body be checked by such causes."

M. Morel considers the phenomena of degeneration, as existing in cretinous districts, as eminently allied to these, and as arising from similar causes, most especially from a quasi-marshy geological structure. He states that it is, in Savoy, almost exclusively on argillaceous soils, and those of chalky clay, that goitrous and cretinous affections appear. Wherever we meet with hills formed of *clay schist*, or declivities of a black glutinous earth in which the rain torrents dig deep trenches, or enormous deposits of gypsum, there we may be certain of finding people profoundly affected with goitre and cretinism.

Monseigneur Billiet, Archbishop of Chambery, views the

* By Mr. Horne.

causes as primary and secondary. "I regard," says he, "as *secondary causes*, hygienic conditions, the external configuration of the soil, the narrowness of the valleys, excessive humidity, and the bad construction and dirt of the habitations. . . But the *primary cause* must be sought, not in the exterior configuration of the country, but in its mineralogical structure. These affections are endemic, because the population has fixed its abode in the country which produces them. The localities which now possess them have always had, will always have, them. Take this population into a healthy locality, and after one or two generations they will be free from these infirmities. That which replaces it will in due time become affected in the same manner, because the true cause of the evil is neither in hygienic conditions nor in the blood of the people ; *it is under the surface of the soil—not above it.* The soil exerts its influence upon the people by its effects upon the water, and, perhaps, the produce of the earth. What is the mineral which produces these effects ? Is it magnesia, as M. Grange suggests ; or the absence of iodine, as according to M. Chatin ?"

M. Morel agrees generally with these views, but is of opinion that " cretinism may be extirpated from a country without resorting to the desperate expedient of quitting it," and appeals for confirmation to the beneficial results to be obtained in analogous degenerations in marshy districts. Considering primarily the cause of cretinism and allied affections to be a poisonous influence exerted by miasm upon the cerebro-spinal system, he still allows due weight to the over-abundance of magnesia and the absence of iodine ; and adopts a system of therapeutics applicable to all theories.

> "We seek to separate those who are threatened or attacked from the infected locality. We place them in elevated districts where they breathe a purer air. We modify the nature of the water by adding iodine in some form. We seek to fortify the enfeebled constitution ; we administer tonics, and strengthening baths ; we act upon the nervous system by means of electricity ; we employ gymnastics ; we seek by every means to awaken the senses. We have the greatest confidence in the influence of the moral upon the physical nature. We make energetic appeals to what remains of emotion and intelligence, to arrest the march of the evil, and to prevent complete degeneration. This is not an empty theory, but one the effects of which have been proved in successful practice. But we attack also the evil in its *origin and source ;* we render the localities wholesome by embanking rivers, and converting stagnant pools into running water. By these and such measures, cretinism has been extirpated at Robestsau near Strasbourg, and other localities. The complete disappearance of the evil depends on *collective effort* in drying and draining morasses ; in isolating those persons who are likely to propagate it hereditarily ; and in giving proper moral and intellectual culture in the schools or districts where the evil exists."

These curative elements are of more extended importance, inasmuch as the cretinous development is always found, more or less closely socially allied to other affections of an eminently degenerative nature. Goitre, deaf-dumbness, rachitis, imbecility and idiocy, scrofulous and tuberculous affections, hernia, chronic gastritis; all these exist concurrently with cretinism, are due to like causes, and are necessarily amenable to the same hygienic, prophylactic and therapeutic agencies.

In the atlas of plates accompanying M. Morel's valuable work, there are illustrations of the typical forms of degeneration arising from the various sources already mentioned; as those from alcoholism, from the geological structure of the soil, from malnutrition, from cretinism, &c. There is one series so illustrative of the author's views of *progressive* degeneration, and so instructive in all respects, as to demand some detailed notice. It represents the father and mother of a family, with their six children, born during a space of twelve years, during which the progress of degradation is traceable in the most interesting and conclusive manner. The district is that of la Meurthe.

Marie, the mother, aged 54, has had eleven children, only six survive. She became goitrous at 30 years of age. She has not the cretinous type, *but is emaciated and aged from privation and misery.* Her intelligence is normal, and there have been no cretins in her family.

Joseph, the father, is goitrous from birth; he is the sole survivor of his father's family; his father and grandfather were semi-cretins, as he is. His head is flattened posteriorly, and *very wide in the bilateral diameter.* His intellect is feeble.

Annette, aged twenty-six, is slow in locomotion, and rather torpid in intelligence—slightly goitrous. Otherwise she is normal, physically and morally. She has two young children, in whom, *as yet*, no sign of degeneration is apparent, except precocious *etiolation* due to misery and want.

Rosine, aged twenty-four, bears further physiognomical marks of deterioration, in the nearer approach to the cretinous feature. Her intelligence is obtuse, and her moral sense very defective. She has a natural child, *as yet* healthy.

Ireneus, aged twenty-two, presents a still further advance in intellectual and physical decay. He has a *puffy* figure, badly-formed head, and is below medium stature. His genital organs are enormous, but unfit for reproduction. He has the cretin temperament, without the precise typical form. He cannot be taught to read or write, nor to perform any useful work—he is also deaf.

Pauline, aged seventeen, is congenitally deaf and dumb. She is goitrous, and utterly undeveloped morally and intellectually. Her actions are automatic, and her character *sad*, but irritable. There is a general arrest of development physically.

Françoise, aged sixteen, and Agnes, aged fifteen, are perfect cretins; the arrest of development is irremediable; there is no sign of puberty, and they will remain during life what they now are, completely degenerate beings. They cannot speak, and scarce can walk. Their skin is yellow, and the flesh œdematous. Dentition was very irregular, and some of the first teeth still remain.

The children born after this period all died; reproduction had reached its term.

In conclusion M. Morel briefly reviews the ground already passed over, and sketches the plan which he proposes to himself for the forthcoming work (intended as a continuation to the present one) on hygiene and therapeutics. Viewing mental alienation as the last term of a series of degenerations, he continues:—

"If the idea which I have formed of the moral, intellectual, and physical causes of this degeneration be correct, the plan which I must follow in my curative indications arises naturally from these preliminary investigations. Moral and physical hygiene; the treatment of the acute stage; prophylaxis; these are the three terms which best represent the fundamental therapeutic questions to be elucidated."

1. The moral treatment, which is but the application of the great duties imposed by the divine moral law, fixed and immutable, is not, as M. Morel states, a new thing; but what is new is the precise enunciation of the special data which must preside over this class of treatment, "in regard to temperament, age, hereditary predisposition, and all the morbid organic conditions, intellectual and physical, which constitute states of suffering—in other words, *degenerations*."

"The moral law certainly is *one* and universal; and the possibility of its being accepted and practised by all, is one of the strongest presumptions in favour of the unity of the species.

"But races have not all arrived at the same degree of civilization; and in the midst of civilized nations themselves, there exist *degenerate races*, who have scarce a perception of the progress of the superior classes, *and who cannot attain to its benefits*, if left to their own unaided resources. These are the victims of the '*mixed causes;*' and the application of the moral treatment to these disinherited masses, is at once one of the noblest and most difficult studies which can engage the true friends of humanity. I shall tell nothing new to physicians in saying that physical is inseparable from moral hygiene; but *there are moralists who have need to be convinced that the moral law can only become fruitful in a sound organism.*"

2. All the causes of degeneration, already reviewed, produce profound effects, first upon individuals, and through them upon races or communities. M. Morel considers that there is no form of degeneration whatever (mental alienation included), which has not its *acute* stage or period, during which the resources of medicine may be expected to be attended with success, *if applied in time.* This is the ground where medical science appears to most advantage; but "the duty of the physician does not cease with the transitory cares bestowed upon acute or accidental diseases."

3. There is another sphere where his science and devotion are shown, perhaps less brilliantly, but in a manner more conducive to the general and ultimate good of humanity. This is in prophylaxis—in other words, the science which proposes for its end and aim to combat the causes of disease and prevent their effects.

Prophylaxis is of two kinds, *defensive* and *preservative.* The removal of criminals and persons of unsound mind from general society into prisons and asylums is of the former kind; inasmuch as society is thereby, and *for the time*, defended from their evil influence. But the period of this form of prophylaxis is limited in the one case by the term of punishment, and in the other, by the date of apparent cure. "Both enter society again, and it is to these that the application of the moral law must be made in its most extended sense. Society must adopt then a preservative prophylaxis by attempting to modify the moral, physical, and intellectual conditions of those who have been separated from them: before restoring them to their former social position, they must be armed against themselves, in order to lessen the number of relapses."

We cannot more aptly conclude our analysis of Morel's able and philosophical work than in his own words:—

"The plan which I have adopted is vast; but I am determined to pursue it to the end. The confidence which sustains me does not arise from any exaggerated idea of my own strength, but from a lively and profound faith which strengthens and animates me. I believe that the study of the causes of degeneration, and of their treatment, is one of the most important, useful, and suggestive that can occupy the mind of a physician; and that it is the duty of each, according to his power and ability, to aid in preventing the generalization of the evils pointed out; and thus to have ever before him, as the programme of his labours, the *intellectual, moral, and physical amelioration of man*, or (if the term be preferred) HIS REGENERATION."

